# Assessment of Relationship of Ketamine Dose With Magnetic Resonance Spectroscopy of Glx and GABA Responses in Adults With Major Depression: A Randomized Clinical Trial

**DOI:** 10.1001/jamanetworkopen.2020.13211

**Published:** 2020-08-12

**Authors:** Matthew S. Milak, Rain Rashid, Zhengchao Dong, Lawrence S. Kegeles, Michael F. Grunebaum, R. Todd Ogden, Xuejing Lin, Stephanie T. Mulhern, Raymond F. Suckow, Thomas B. Cooper, John G. Keilp, Xiangling Mao, Dikoma C. Shungu, J. John Mann

**Affiliations:** 1 Department of Psychiatry, College of Physicians and Surgeons, Columbia University, New York, New York; 2 Molecular Imaging and Neuropathology Division, The New York State Psychiatric Institute, New York; 3 Department of Radiology, College of Physicians and Surgeons, Columbia University, New York, New York; 4 Department of Biostatistics, Mailman School of Public Health, Columbia University, New York, New York; 5 Analytical Psychopharmacology Laboratory, The Nathan S. Kline Institute for Psychiatric Research, Orangeburg, New York; 6 Department of Radiology, Weill Cornell Medicine, New York, New York

## Abstract

**Importance:**

A single subanesthetic dose of ketamine produces an antidepressant response in patients with major depressive disorder (MDD) within hours, but the mechanism of antidepressant effect is uncertain.

**Objective:**

To evaluate whether ketamine dose and brain glutamate and glutamine (Glx) and γ-aminobutyric acid (GABA) level responses to ketamine are related to antidepressant benefit and adverse effects.

**Design, Setting, and Participants:**

This randomized, parallel-group, triple-masked clinical trial included 38 physically healthy, psychotropic medication–free adult outpatients who were in a major depressive episode of MDD but not actively suicidal. The trial was conducted at Columbia University Medical Center. Data were collected from February 2012 to May 2015. Data analysis was conducted from January to March 2020.

**Intervention:**

Participants received 1 dose of placebo or ketamine (0.1, 0.2, 0.3, 0.4, or 0.5 mg/kg) intravenously during 40 minutes of a proton magnetic resonance spectroscopy scan that measured ventro-medial prefrontal cortex Glx and GABA levels in 13-minute data frames.

**Main Outcomes and Measures:**

Clinical improvement was measured using a 22-item version of the Hamilton Depression Rating Scale (HDRS-22) 24 hours after ketamine was administered. Ketamine and metabolite blood levels were measured after the scan.

**Results:**

A total of 38 individuals participated in the study, with a mean (SD) age of 38.6 (11.2) years, 23 (60.5%) women, and 25 (65.8%) White patients. Improvement in HDRS-22 score at 24 hours correlated positively with ketamine dose (*t*_36_ = 2.81; *P* = .008; slope estimate, 19.80 [95% CI, 5.49 to 34.11]) and blood level (*t*_36_ = 2.25; *P* = .03; slope estimate, 0.070 [95% CI, 0.007 to 0.133]). The lower the Glx response, the better the antidepressant response (*t*_33_ = −2.400; *P* = .02; slope estimate, −9.85 [95% CI, −18.2 to −1.50]). Although GABA levels correlated with Glx (*t*_33_ = 8.117; *P* < .001; slope estimate, 0.510 [95% CI, 0.382 to 0.638]), GABA response did not correlate with antidepressant effect. When both ketamine dose and Glx response were included in a mediation analysis model, ketamine dose was no longer associated with antidepressant effect, indicating that Glx response mediated the relationship. Adverse effects were related to blood levels in men only (*t*_5_ = 2.606; *P* = .048; estimated slope, 0.093 [95% CI, 0.001 to 0.186]), but Glx and GABA response were not related to adverse effects.

**Conclusions and Relevance:**

In this study, intravenous ketamine dose and blood levels correlated positively with antidepressant response. The Glx response correlated inversely with ketamine dose and with antidepressant effect. Future studies are needed to determine whether the relationship between Glx level and antidepressant effect is due to glutamate or glutamine.

**Trial Registration:**

ClinicalTrials.gov Identifier: NCT01558063

## Key Points

**Question:**

What is the relationship between the antidepressant effect of ketamine and ketamine dose and blood level, and is its antidepressant effect mediated by an effect on ventro-medial prefrontal cortical glutamate and glutamine or γ-aminobutyric acid response?

**Findings:**

This randomized clinical trial of 38 patients with major depression found a relationship of ketamine dose and blood level with antidepressant response at 24 hours. Ketamine suppression of glutamate and glutamine in the ventro-medial prefrontal cortex mediated the relationship of ketamine dose and level with antidepressant effect but was unrelated to psychotomimetic side effects.

**Meaning:**

The findings of this study suggest that glutamate and glutamine suppression by ketamine may be a potential biomarker of rapid antidepressant effect and that a fast-acting antidepressant without psychotomimetic adverse effects may be possible.

## Teaser

This randomized clinical trial evaluates whether ketamine dose and brain glutamate and glutamine (Glx) and γ-aminobutyric acid (GABA) level responses to ketamine are related to antidepressant benefit and adverse effects.

## Introduction

Major depressive disorder (MDD), a leading cause of disability worldwide,^1^ affects more than 16 million adults in the United States,^2^ with estimated costs of $210.5 billion in 2010, a 21.5% increase from 2005.^3^ Response to currently marketed antidepressants generally requires treatment for 6 to 8 weeks,^4^ and they are ineffective in 30% to 50% of patients.^5,6^ Faster-acting and more effective antidepressants are needed.

A single intravenous (IV) subanesthetic dose of ketamine can produce an antidepressant response in hours instead of weeks, even in medication-resistant MDD.^7,8,9,10,11,12,13,14,15,16^ Ketamine has antidepressant benefit in both patients with MDD^17,18^ and those with bipolar depression.^19^ Adverse effects of ketamine include transient depersonalization and derealization, among other psychotomimetic and dissociative symptoms.^20,21^ Understanding the mechanism of its rapid-onset antidepressant action may aid identification of alternative medications that can be used orally, have fewer adverse effects, and have less abuse potential.

We previously reported on an open pilot MDD study that showed, consistent with glutamate increases in rodent studies,^22,23^ that ketamine induces an acute increase in ventro-medial prefrontal cortex (mPFC) levels of the combined resonance of glutamate and glutamine (Glx), measured with proton magnetic resonance spectroscopy (1H MRS).^24^ Unexpectedly, we also observed an increase in γ-aminobutyric acid (GABA) levels that correlated positively with the increase in Glx levels. Preclinical studies suggest that ketamine’s antidepressant mechanism of action may be mediated by glutamate activation of the glutamatergic α-amino-3-hydroxy-5-methyl-4-isoxazolepropionic acid (AMPA) receptors^25,26,27,28^ and the downstream induction of the neurotrophin^29,30^ and mammalian target of rapamycin^29,30^ (mTOR; also known as mechanistic TOR) signaling pathways. An alternative model^31,32^ suggests that it is not glutamate that mediates ketamine’s antidepressant action; rather, it is a direct consequence of ketamine's inhibitory effect on the N-methyl-D-aspartate (NMDA) receptors. The role of GABA is unknown, but an increase in its level reverses the reported GABA deficit in major depression.^33,34,35,36,37^

In the current study, we sought to determine whether the dose of ketamine or blood levels of ketamine and its metabolites are correlated with antidepressant effect and adverse effects. We also explored whether ketamine or metabolite levels correlate with mPFC Glx or GABA levels and, in turn, whether Glx or GABA levels mediate the acute antidepressant or adverse effects of ketamine among individuals with MDD. To do this, we conducted a randomized, placebo-controlled, dose-finding, clinical trial in patients with MDD not currently receiving medication, who underwent MRS measurements of mPFC Glx and GABA during the IV administration of ketamine. Blood levels of ketamine and metabolites were assayed after the scan.

## Methods

### Patients

**Figure 1.**
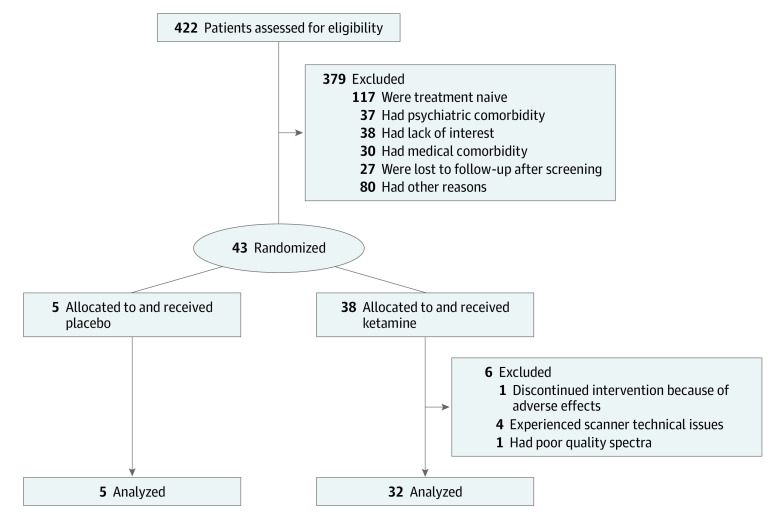
Study Flow Diagram

Figure 1 shows the study flow. Of the 43 patients, 5 were unable to complete the study because of scanner-related discomfort or technical issues. For the final analysis, we included 38 physically healthy patients, aged 18 to 59 years, who met the *Diagnostic and Statistical Manual of Mental Disorders* (Fourth Edition) criteria for a major depressive episode (MDE) in the context of MDD and scored at least 22 on the Montgomery-Åsberg Depression Rating Scale (Table), which was only used to establish depression severity for determining eligibility to prevent inflation of baseline scores^38,39,40^ on the primary outcome measure (a 22-item version of Hamilton Depression Rating Scale [HDRS-22]). Patients were not taking any psychotropic medications and had not been taking medications likely to interact with GABA or glutamate for at least 14 days, neuroleptics for at least 1 month, or fluoxetine for at least 6 weeks before receiving ketamine. The detailed protocol is available in Supplement 1.

**Table.** Demographic and Clinical Characteristics of Participants by Assigned Intravenous Ketamine Dose

Characteristic	Mean (SD), by dose group						Statistical differences	
	0.0 mg/kg (n = 5)	0.1 mg/kg (n = 5)	0.2 mg/kg (n = 6)	0.3 mg/kg (n = 8)	0.4 mg/kg (n = 5)	0.5 mg/kg (n = 9)	*F*_5,32_	*P* value
Age, y	46.8 (12.3)	37.4 (12.3)	37.8 (8.2)	38.1 (7.2)	30.6 (9.4)	40.2 (14.5)	1.119	.37^a^
Women, No. (%)	2 (40)	4 (80)	5 (83)	4 (50)	3 (60)	5 (56)	NA	.69^b^
White race, No. (%)	4 (80)	1 (20)	4 (67)	7 (88)	2 (40)	7 (78)	NA	.14^b^
Hispanic ethnic group, No. (%)	1 (20)	1 (20)	1 (17)	0 (0)	1 (20)	2 (22)	NA	.84^b^
Age of MDD first episode onset, y	28.8 (16.9)	20.6 (10.3)	13.3 (2.7)	20.6 (8.2)	19.4 (5.6)	16.6 (8.1)	1.778	.15^a^
Duration of current MDE, y	5.3 (3.1)	4.1 (4.4)	13.7 (15.9)	8.1 (12.5)	8.8 (11.2)	16.2 (19.7)	0.790	.57^a^
Duration of MDD, y	18.0 (14.7)	16.8 (14.4)	24.6 (9.6)	17.3 (11.6)	10.9 (8.8)	23.7 (16.6)	0.876	.51^a^
Previous MDEs, No.	0.4 (0.5)	1.0 (1.4)	1.7 (1.9)	1.5 (2.1)	1.0 (1.4)	0.8 (0.8)	0.609	.69^a^
Baseline MADRS total score	32.8 (4.9)	31.0 (6.3)	28.0 (3.6)	31.1 (3.0)	32.8 (1.3)	30.9 (4.1)	0.935^c^	.47^a^
Baseline HDRS-22 total score	25.0 (3.4)	25.0 (5.5)	24.2 (4.3)	25.9 (7.7)	27.2 (5.0)	27.0 (5.3)	0.290	.93^a^
Change in HDRS-22 total score 24 h after ketamine infusion, %	−2.8 (3.7)	−6.2 (13.0)	−6.7 (7.3)	−8.3 (6.3)	−10.8 (6.8)	−13.3 (7.9)	NA	NA
AUC blood level, ng/mL^d^								
Ketamine	0.0 (0.0)	27.2 (7.0)	44.5 (18.9)	59.1 (12.2)	71.6 (16.8)	112.8 (27.9)	NA	NA
Norketamine	0.0 (0.0)	30.2 (17.2)	31.8 (6.8)	56.6 (18.4)	71.0 (21.8)	95.2 (25.6)	NA	NA
Dehydronorketamine	0.0 (0.0)	17.6 (4.8)	31.8 (11.7)	39.9 (9.2)	42.8 (9.6)	52.1 (17.5)	NA	NA

Abbreviations: AUC, area under the curve; HDRS-22, 22-item Hamilton Depression Rating Scale; MADRS, Montgomery-Åsberg Depression Rating Scale; MDD, major depressive disorder; MDE, major depressive episode; NA, not applicable.

^a^ Results of between groups analysis of variance.

^b^ Result of Fisher exact test.

^c^ Result for *F*_5,31_ statistic.

^d^ Area under the curve calculated for ketamine and metabolite blood levels as the sum of their respective blood levels at 90 and 120 minutes postinitiation of ketamine infusion.

Exclusion criteria included lifetime history of bipolar disorder, schizoaffective disorder, schizophrenia, or any other psychotic disorder, including MDD with psychotic features; a first-degree relative with bipolar disorder, schizoaffective disorder, or schizophrenia, with the potential participant younger than 33 years (ie, still at age of risk for a psychotic disorder); receipt of electroconvulsive therapy within 3 months of enrolling in the study; history of IV drug use; nonresponse or intolerance to ketamine (defined as participation in another ketamine study and reporting to us less than a robust response); pregnancy, planning to conceive, or sexually active but not using adequate birth control; and contraindications to magnetic resonance imaging (MRI). Patients who were actively suicidal were also excluded. All patients were outpatients. All patients were enrolled after a psychiatric and medical screening, conducted between February 2012 through May 2015, determined that they met the entrance criteria and after they provided written informed consent. The study was approved by the New York State Psychiatric Institute institutional review board and was completed in October 2019, before recruitment goals were met (see details in Study Design section). This study followed the Consolidated Standards of Reporting Trials (CONSORT) reporting guideline.

### Study Design

Randomization and assignment of ketamine dose (ie, 0.1, 0.2, 0.3, 0.4 or 0.5 mg/kg) or placebo were performed by the statistician. An adaptive randomization strategy was used to optimize group size in terms of dose response curves. The randomization was supposed to have 11 participants per group, but the study was stopped before recruiting goals were met because funding ran out and an interim analysis found robust statistical effects. Randomized ketamine doses were shared via sealed nontranslucent envelopes with the research pharmacy and a clinician who was not involved in patient study ratings. Study patients and raters were masked until study termination unless unmasking was clinically warranted. No patient required unmasking in the course of the ketamine infusion. Patients received a single 40-minute infusion and were observed for 24 hours after ketamine administration. All scans and data were obtained at Columbia University Medical Center.

Prior to treatment infusion, patients underwent structural MRI and baseline 1H MRS scans. Scan analysis was performed on coded data sets masked to the subject dosing and treatment response. Patients were administered placebo or ketamine intravenously during approximately 40 minutes. Six 1H MRS data frames of approximately 13 minutes each were acquired: 1 prior to ketamine infusion and 5 during and immediately after ketamine infusion. We determined clinical response 24 hours after ketamine administration using the HDRS-22. The masking procedures and randomization ensured minimal possibility of biasing based on Cochrane criteria.^41^

### Safety and Tolerability

Vital signs, including blood pressure and heart rate, were monitored 5 minutes prior to ketamine infusion, every 5 minutes during the 40-minute ketamine infusion, and every 5 minutes after for the duration of the 1H MRS scan or until vital signs returned to clinically acceptable levels. Patients were evaluated by a physician for blood pressure, psychosis and other psychotomimetic adverse effects (using the Brief Psychiatric Rating Scale [BRPS]), and suicidal ideation (using the Columbia–Suicide Severity Rating Scale) 230 minutes after the initiation of the ketamine infusion. Four serious adverse events occurring during study participation were reported to the institutional review board: 1 for suicide, 1 for active suicidal ideation, 1 for antidepressant misuse, 1 for unrelated medical illness (see eTable in Supplement 2).

### Pharmacokinetic Assessments

Blood samples were collected at 90 and 120 minutes after initiation of ketamine infusion to assay plasma concentrations of ketamine and metabolites.^24^ Blood samples were not collected for technical reasons during the approximately 80-minute 1H MRS acquisition. We used the sum of the 2 ketamine and metabolite levels to get a more stable measurement of their plasma concentrations across participants because ketamine is metabolized rapidly and variably.^42^

### Clinical Assessments

The 24-item HDRS was administered within 60 minutes before, 230 minutes after, and 24 hours after initiation of ketamine infusion. We modified the 24-item HDRS, excluding items 16 (loss of weight) and 18 (diurnal variation) because a change in these measures could not be assessed within 24 hours. Response was defined as percentage change on this 22-item HDRS 24 hours after ketamine administration.

### Magnetic Resonance Neuroimaging Scans

All the neuroimaging data were acquired on a GE Signa EXCITE 3.0T MR scanner equipped with an 8-channel surface coil, as previously described.^24^ Briefly, a 3-plane localizer imaging series was obtained, followed by a 2-dimensional fast spoiled gradient-recalled echo MRI scan in sagittal planes (echo time, 2.1 milliseconds; repetition time, 75 milliseconds; flip angle, 75°; field of view, 256 × 256 mm^2^; slice thickness, 5 mm; 8 slices) and a volumetric T_1_-weighted spoiled gradient-recalled MRI scan prescribed in the oblique axial planes parallel to the anterior commissure–posterior commissure line (echo time, 2.86 milliseconds; repetition time, 7.12 milliseconds; flip angle, 9°; field of view, 256 × 256 mm^2^; image matrix size, 256 × 256; slice thickness, 1 mm; voxel size, 1 × 1 × 1 mm^3^). A voxel of 3.0 × 2.5 × 2.5 cm^3^ was placed in the ventral mPFC region based on the sagittal and oblique axial images, with the center of the posterior side of the voxel close to the front tip of the cingulate gyrus (eFigures 1A and 1B in Supplement 2). In vivo brain spectra of the combined resonance of Glx and GABA were recorded from the voxel using the standard J-edited spin echo difference method.^43,44^ Data were acquired as six 13-minute frames. The levels of Glx and GABA in the edited spectra were fitted in the frequency domain as previously described (eFigure 1C in Supplement 2)^43,44^ and then expressed as peak area ratio relative to the synchronously acquired and similarly fitted unsuppressed voxel water signal—a commonly used^45,46,47,48,49^ method with reasonable test-retest reliability.^44^ Data from 1 participant were excluded from further analysis because of poor-quality spectra. Details of the 1H MRS data quality assessment criteria used to retain or reject spectra for inclusion in group analyses can be found in previously published supplemental online material.^44^

### Statistical Analysis

Ketamine, norketamine, and dehydronorketamine blood levels (ng/mL) were measured as the mean of the levels at 90 and 120 minutes. Levels of Glx and GABA in the mPFC were measured as the area under the curve from 0 to approximately 42 minutes following initiation of ketamine infusion. Clinical improvement was measured as the percentage change in HDRS-22 score from baseline to 24 hours after dose. Psychotomimetic effects were measured with the BPRS total score.

To examine the effect of ketamine dose on clinical improvement, we fit a simple linear regression model. Next, we fit separate simple linear regression models to test the effect of ketamine dose on ketamine blood level, Glx level, and GABA level. With similar models, we explored the effect of ketamine blood level on Glx level and on GABA level. Next, we fit separate simple linear regression models with clinical improvement as outcome and ketamine blood level, Glx level, and GABA level as independent variables. Finally, to determine whether Glx level mediates the effect of ketamine dose on clinical improvement, we also fit a multiple regression model with clinical improvement as outcome and both ketamine dose and Glx level as independent variables.^50,51,52^ For each analysis we reported the test statistic value with degrees of freedom, *P* value, and parameter estimate with its 95% CI.

In the analysis of the adverse effects data, we fit separate simple linear regression models with psychotomimetic effects as outcome and as predictors: blood ketamine level, Glx, GABA, norketamine, dehydronorketamine, and clinical improvement. These analyses were repeated separately for men and women. Data analysis was conducted in R version 3.6.3 (R Project for Statistical Computing) from January to March 2020. Statistical significance was set at *P* < .05, and all test were 2-tailed but not corrected for multiple comparisons over the entire analytic set.

## Results

A total of 38 individuals participated in the study, with a mean (SD) age of 38.6 (11.2) years, 23 (60.5%) women, and 25 (65.8%) White patients. The Table describes the patient population demographic characteristics, depression severity, and clinical response to each ketamine dose. The ketamine dose and placebo treatment groups did not differ statistically in demographic characteristics, baseline depression severity or duration of current episode, number of lifetime episodes, or years since onset of MDD. Notably, the entire patient sample had a long duration of their current major depressive episode.

### Effects of Ketamine IV Dose and Blood Level on Clinical Improvement

**Figure 2.**
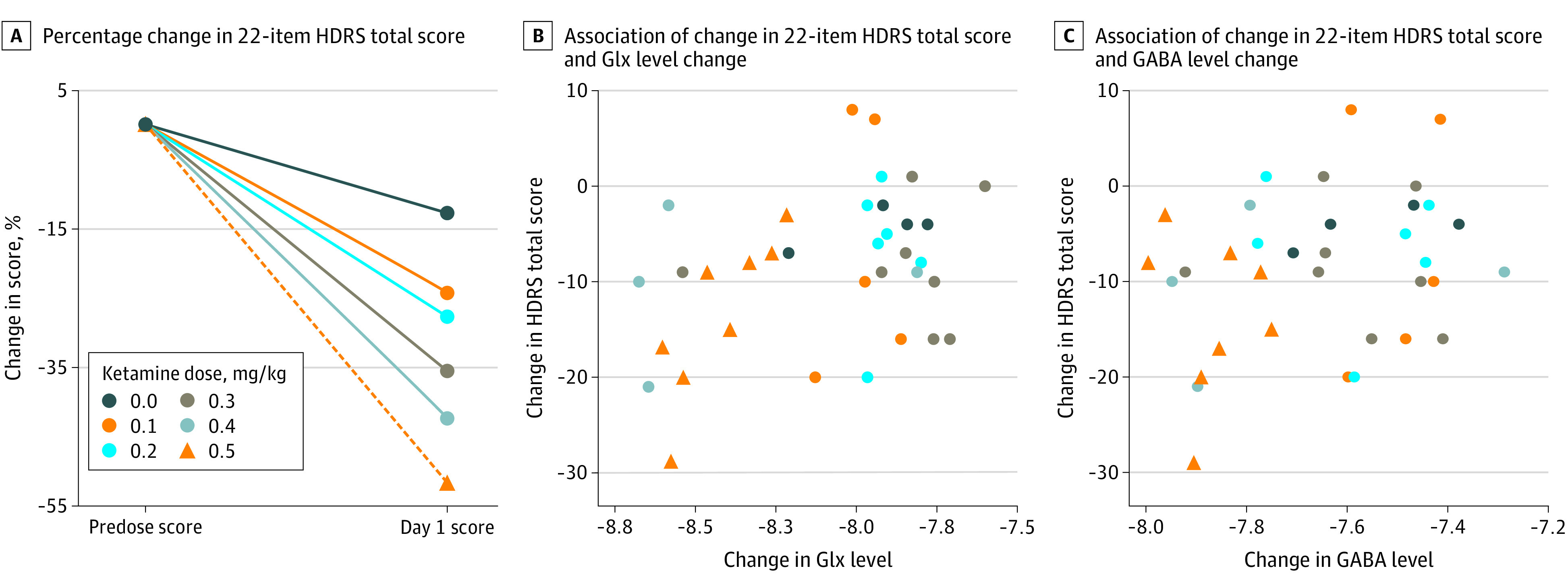
Changes in Modified 22-Item Hamilton Depression Rating Scale (HDRS) Score 24 Hours After Ketamine Intravenous Dose GABA indicates γ-aminobutyric acid; Glx, glutamate and glutamine.

Ketamine dose effects on clinical improvement are summarized in the Table and Figure 2A. Injected dose of ketamine had a positive relationship with clinical improvement (*t*_36_ = 2.81; *P* = .008; slope estimate, 19.80 [95% CI, 5.49 to 34.11]), as did the ketamine blood level (*t*_36_ = 2.25; *P* = .03; slope estimate, 0.070 [95% CI, 0.007 to 0.133]). Metabolite levels did not correlate statistically with clinical improvement (norketamine: *t*_35_ = 1.88; *P* = .07; slope estimate, 0.07 [95% CI, −0.006 to 0.146]; dehydronorketamine: *t*_35_ = 1.13; *P* = .27; slope estimate, 0.075 [95% CI, −0.060 to 0.211]).

### Relationship of Ketamine Dose With Blood Level

Ketamine dose correlated positively with ketamine blood level (*t*_36_ = 12.08; *P* < .001; slope estimate, 10.5 [95% CI, 175.2 to 245.9]). The results of this test are illustrated in eFigure 2 in Supplement 2.

### Effects of Ketamine Blood Level on Glx and GABA Levels

**Figure 3.**
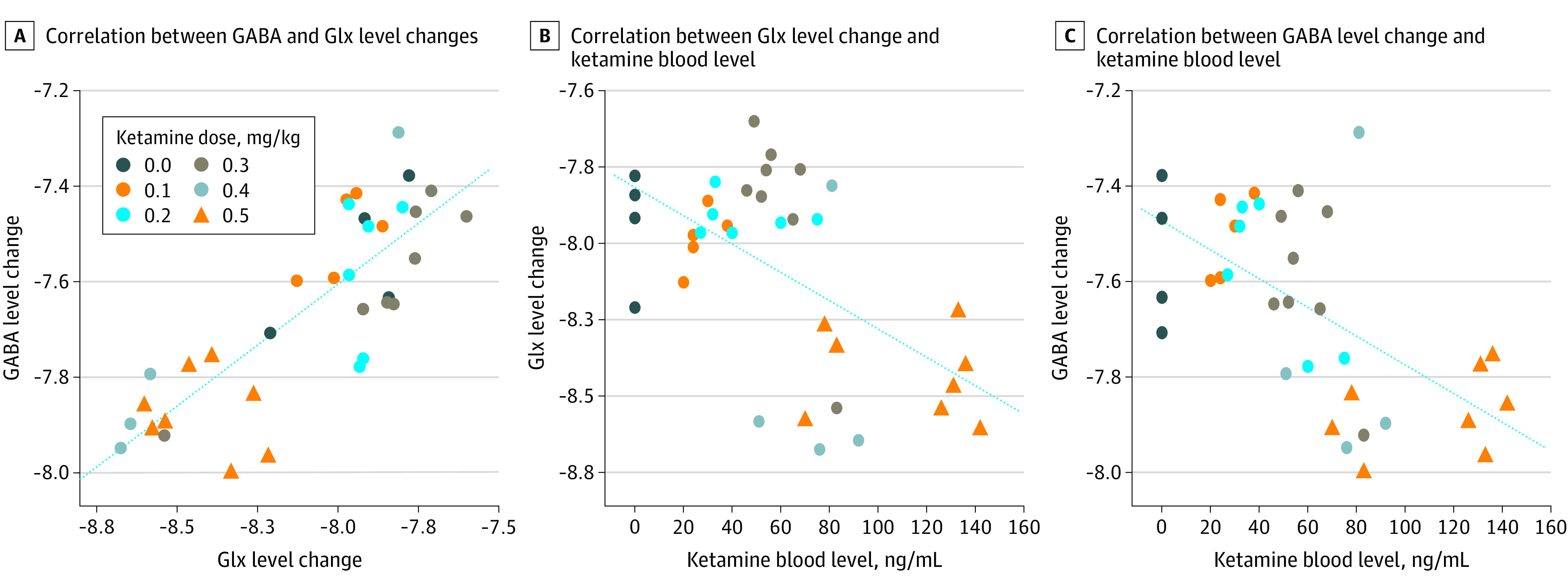
Correlations Between Glutamate and Glutamine (Glx), γ-Aminobutyric Acid (GABA), and Ketamine Blood Levels Level change was measured as area under the curve 0 to approximately 42 minutes following initiation of ketamine infusion.

The injected dose of ketamine showed a negative relationship with Glx (*t*_33_ = −4.120; *P* < .001; slope estimate, −1.088 [95% CI, −1.626 to −0.551]) and, in a separate model, with GABA (*t*_33_ = −4.450; *P* < .001; slope estimate, −0.714 [95% CI, −1.041 to −0.388]). The ketamine blood level also had a negative relationship with Glx (*t*_33_ = −4.087; *P* < .001; slope estimate, −0.005 [95% CI, −0.007 to −0.002]) (Figure 3B) and GABA (*t*_33_ = −4.334; *P* < .001; slope estimate, −0.003 [95% CI, −0.004 to −0.002]) (Figure 3C).

### Effects of Glx and GABA on Clinical Improvement

We found a positive relationship between Glx and clinical improvement (*t*_33_ = −2.400; *P* = .02; slope estimate, −9.85 [95% CI, −18.2 to −1.50]) (Figure 2B). When both ketamine blood level and Glx were used as predictors in a multiple regression analysis, neither the effect of Glx on clinical improvement (*t*_32_ = −1.417; *P* = .17; slope estimate, −7.153 [95% CI, −17.436 to 3.130]) nor the effect of ketamine blood level on clinical improvement (*t*_32_ = 0.922; *P* = .36; slope estimate, 0.037 [95% CI, −0.045 to 0.119]) remained significant. Similarly, when both injected ketamine dose and Glx were used in a multiple regression analysis, neither the effect of Glx on clinical improvement (*t*_32_ = −1.103; *P* = .28; slope estimate, −5.465 [95% CI, −15.554 to 4.624]) nor the effect of injected ketamine dose on clinical improvement (*t*_32_ = 1.519; *P* = .14; slope estimate, 14.053 [95% CI, −4.787 to 32.893]) remained significant. Although Glx had a significant positive relationship with GABA (*t*_33_ = 8.117; *P* < .001; slope estimate, 0.510 [95% CI, 0.382 to 0.638]) (Figure 3A), GABA was not associated with clinical improvement (*t*_33_ = −1.552; *P* = .13; slope estimate, −10.67 [95% CI, −24.66 to 3.32]) (Figure 2C).

### Adverse Effects

Psychotomimetic effects were measured with the BPRS. Ketamine blood level was not related to BPRS score when analyzing the full sample (*t*_23_ = 1.084; *P* = .29; estimated slope, 0.29 [95% CI, −0.26 to 0.084]). In men only, ketamine blood level had a positive relationship with BPRS score (*t*_5_ = 2.606; *P* = .048; estimated slope, 0.093 [95% CI, 0.001 to 0.186]), while there was no effect in women (*t*_16_ = −0.362; *P* = .72; estimated slope, −0.013 [95% CI, −0.086 to 0.061]). In men, both norketamine and dehydronorketamine blood levels had a positive relationship with BPRS score (norketamine: *t*_5_ = 3.944; *P* = .01; estimated slope, 0.139 [95% CI, 0.049 to 0.230]; dehydronoketamine: *t*_5_ = 2.589; *P* = .049; estimated slope, 0.167 [95% CI, 0.001 to 0.334]).

We did not find a correlation between Glx response and BPRS score in the full sample (*t*_22_ = −0.015; *P* = .99; estimated slope, −0.054 [95% CI, −7.35 to 7.24]), in men only (*t*_5_ = −0.051; *P* = .96; estimated slope, −0.401 [95% CI, −20.42 to 19.62]), or in women only (*t*_15_ = 0.156; *P* = .88; estimated slope, 0.630 [95% CI, −7.98 to 9.24]). GABA did not correlate with BPRS score (data not shown).

Clinical improvement was not related to BPRS score (*t*_23_ = −1.01; *P* = .32; estimated slope, −0.32 [95% CI, −0.97 to 0.34]). Other significant adverse effects, including vomiting, rise in blood pressure requiring intervention, and so on, did not occur.

## Discussion

In this study, we found a positive correlation of ketamine IV dose and blood level with antidepressant effect 24 hours after ketamine administration. We also observed that ketamine produced a dose-dependent decrease in mPFC Glx level and that a lower mean Glx level was associated with better antidepressant response. Although Glx and GABA levels were correlated, the latter was unrelated to antidepressant effect, and both were not related to psychotomimetic adverse effects. Psychotomimetic side effects correlated with blood levels of ketamine in men only. Metabolites did not appear to be statistically related to antidepressant effect but were correlated with psychotomimetic adverse effects in men.

### Effect of Ketamine Dose on Clinical Improvement

Our finding that IV ketamine dose correlated positively with improvement in the HDRS-22 score (Figure 2A, Table) is supported by a meta-analysis^53^ of 9 ketamine trials—6 of which used the standard IV dose of 0.5 mg/kg per 40 minutes and 3 of which used lower doses—that found lower doses to be less effective. Our findings are also supported by a multicenter ketamine dose-finding randomized clinical trial in 99 patients with treatment-resistant depression,^54^ which found that 1.0 mg/kg and 0.5 mg/kg doses were effective but 0.1 mg/kg and 0.2 mg/kg doses were not. Although an American Psychological Association consensus statement concluded that there are insufficient data to draw firm conclusions about alternative doses,^21^ our findings suggest otherwise. Doses less than 0.3 mg/kg do not appear to be as effective as higher doses.

There is debate regarding whether ketamine’s (2R,6R)-hydroxynorketamine (HNK) metabolite may be as or more important than ketamine itself in mediating the antidepressant effect.^28,55,56^ We found ketamine blood level correlated with antidepressant response but no statistical relationship for norketamine or dehydronorketamine blood levels and antidepressant response. We did not assay HNK metabolites.

### Effect of Ketamine Dose on Brain Glx and GABA Levels

The effects of ketamine on brain Glx (Figure 3B) and GABA (Figure 3C) levels were dose-dependent. We did not measure glutamate or GABA release but overall brain levels. A positron emission tomography study^57^ measured metabotropic glutamate receptor subtype 5 (mGluR5) receptor binding and found that ketamine lowered tracer binding, an indicator of increased glutamate release or receptor internalization. Although preclinical rodent studies^23,58,59^ have reported a ketamine-induced increase in glutamate overflow using microdialysis, that can be unrelated to overall brain tissue glutamate levels and is not detectable by MRS glutamate level measurement, which cannot distinguish between intracellular and extracellular glutamate levels. It is unclear how ketamine may affect Glx or GABA levels or release, although the mGluR2 receptor may mediate a glutamate-related effect.^60^ Perhaps ketamine blocks autoinhibitory presynaptic NMDA receptors that regulate glutamate release,^23,58,59,61^ and inhibition of this negative feedback loop alters glutamate release and production.^23^ By blocking NMDA receptors on GABAergic neurons, ketamine may also alter the inhibitory activity of GABAergic neurons that project to glutamatergic neurons, leading to less GABA.^23,62^

In the present study, Glx was measured as the combined resonances of glutamate and glutamine at approximately 3.75 ppm in the edited MR spectrum.^63^ Because most of the Glx peak is owing to glutamate as opposed to glutamine, it is less likely that any change in Glx is driven by glutamine, but this remains to be confirmed by future studies. We were unable to replicate the increase in Glx (eFigure 3A in Supplement 2) or GABA (eFigure 3B in Supplement 2) detected in our open pilot study in MDD.^22,23^ The biggest increase in Glx observed in the present study was with placebo, and our original pilot study did not have a placebo group. It is possible that the Glx increase during the scan was a stress response and that increasing doses of ketamine diminish that stress response. This interpretation is supported by the finding that cholecystokinin-induced panic in healthy volunteers raises Glx bilaterally in the anterior cingulate.^64^

### Relationship of Glx and GABA Response to Clinical Improvement

To our knowledge, no previous controlled study has examined the relationship of ketamine-induced Glx and GABA effects to clinical response. In this study, the smaller the increase in Glx, the better the clinical response. Ketamine blockade of NMDA receptors would shift the balance of glutamatergic signaling toward metabotropic glutamate (mGlu) and AMPA/kainate receptors.^62^ This effect fits with preclinical studies that suggest that the antidepressant effects of ketamine are related to the effect of glutamate on the AMPA/kainate and/or some of the mGlu receptors, leading to activation of downstream targets, such as the neurotrophin and mTOR signaling pathways, and resulting in the rapid production of mushroom spines, dendritic arborization and synaptogenesis, perhaps to restore and protect neuronal networks that are deficient or dysfunctional in MDD.^30,65,66^ Preclinical studies have also shown that the ketamine-induced activation of the mTOR pathway and subsequent increase in protein synthesis and synaptogenesis, are associated with reduced adenosine triphosphate to adenosine diphosphate ratio and increased phosphorylated AMP–activated protein kinase levels as well as fewer reactive oxygen species measured as carbonylated or damaged proteins, all of which suggest that there is indeed an increase in energy utilization and mitochondrial energy metabolism.^67^ A 2018 study^68^ failed to find that rapamycin blocked the antidepressant effect of ketamine, so we are unsure of its mechanism of action in patients with depression. Mice studies indicate that ketamine may produce a resilience effect that is manifested over a longer time frame,^69^ and through the attenuation of Glx response, we may be observing the onset of this effect. We did not find GABA to be related to antidepressant effect.

### Effect of Ketamine and Its Metabolites on Psychotomimetic Adverse Effects

In this study, the severity of ketamine-induced psychotomimetic adverse effects was related to plasma concentration of ketamine, norketamine, and dehydronorketamine in men but not in women. There may be a sex-based difference in the way ketamine exerts its psychotomimetic adverse effects. Women may preferentially metabolize ketamine through the hydroxynorketamine pathway, as described in an animal study.^28^ Glx and GABA levels did not correlate with psychotomimetic adverse effects, consistent with the importance of NMDA receptor antagonism in mediating this adverse effect. Because psychomimetic side effect severity is also unrelated to antidepressant efficacy, it may be possible to develop newer, comparably fast-acting antidepressants that lack the psychotomimetic adverse effects of ketamine.

### Limitations

This study has limitations. The study sample was small, but the observed effects were robust. A larger sample size is needed to evaluate sex differences in adverse effects. We did not assay HNK metabolite levels and so cannot comment on a possible relationship to antidepressant response. Glx combines glutamate and glutamine, and future studies should try to measure the separate peaks. Active suicidal ideation was an exclusion criterion, so we could not examine the relationship of dose or MRS indices to suicidal ideation response. Given that this study was performed on medication-free research participants, the results need to be reproduced in a clinical treatment–seeking population.

## Conclusions

This study found a robust relationship between ketamine dose and blood levels with antidepressant response. We found that ketamine attenuation of Glx response mediated the relationship between ketamine dose and antidepressant response. Future research should seek to identify the pharmacological effects responsible for Glx response to ketamine, determining if glutamate response accounts for the Glx mediation of antidepressant response, and clarifying the downstream effects of ketamine. Finding new, comparably fast-acting antidepressants without psychotomimetic effects that can be orally administered may be a realistic goal for future research, given that psychotomimetic adverse effects did not correlate with antidepressant or Glx effects of ketamine.

## Notes

Supplement 1. **Trial Protocol**

Supplement 2. **eFigure 1. **Proton Magnetic Resonance Spectroscopy (1H-MRS) Example Voxel Placement and J-Edited Spectra **eFigure 2. **Plasma Concentration of Ketamine **eFigure 3. **Change in Glx and GABA Levels Following Initiation of Ketamine Infusion, Corrected for Internal Water and Expressed as a Percentage of Preketamine Glx **eTable. **Serious Adverse Events Occurring During Study Participation

Supplement 3. **Data Sharing Statement**
