# Supplementary material for: Assessment of Relationship of Ketamine Dose With Magnetic Resonance Spectroscopy of Glx and GABA Responses in Adults With Major Depression: A Randomized Clinical Trial
Source: JAMA Netw Open. 2020 Aug 12;3(8):e2013211. doi: 10.1001/jamanetworkopen.2020.13211 (PMC7424409; doi:10.1001/jamanetworkopen.2020.13211)
Supplement: Supplement 1. — Trial Protocol [file jamanetwopen-3-e2013211-s001.pdf]

New York State Psychiatric Institute

Institutional Review Board

September 18, 2017

To: Dr. Matthew Milak

From: Dr. Edward Nunes, Chairman, IRB

Subject: **APPROVAL NOTICE**: **CONTINUATION APPROVAL**
EXPEDITED PER 45CFR46.110(b)(1)(f)(8)(c)

Your protocol **#6460** entitled: **THE ANTIDEPRESSANT ACTION OF KETAMINE: BRAIN CHEMISTRY** ACAR/PSF version date 9/13/17 and consent forms (version) have been approved by the New York State Psychiatric Institute - Columbia University Department of Psychiatry Institutional Review Board from **OCTOBER 3, 2017 TO OCTOBER 2, 2018.**

Consent requirements:

X Not applicable: (RECRUITMENT COMPLETED. DATA BEING ANALYZED)

☐ 45CFR46.116(d) waiver or alteration of consent for the telephone screen.

☐ Signature by the person(s) obtaining consent is required to document the consent process.

☐ Documentation of an independent assessment of the participant's capacity to consent is also required.

Approved for recruitment of subjects who lack capacity to consent: ☐ No ☐ Yes

Field Monitoring Requirements: ☐ Routine ☐ Special: ____________________

✓ Only copies of consent documents that are currently approved by the IRB may be used to obtain consent for participation in this study.
✓ A progress report and application for continuing review is required 2 months prior to the expiration date of IRB approval.
✓ Changes to this research may not be initiated without the review and approval of the IRB except when necessary to eliminate immediate hazards to participants.
✓ All serious and/or unanticipated problems or events involving risks to subjects or others must be reported immediately to the IRB. Please refer to the PI-IRB website at http://irb.nyspi.org for Adverse Event Reporting Procedures and additional reporting requirements.

CC: CUMC-IRB (no number assigned)
CU Grants & Contracts (5RO1MH093637-03)
RFMH

EN/ls

Signed copy on file at IRB

Protocol Title:
The Antidepressant Action of Ketamine: Brain Chemistry

Version Date:
09/18/2017

Protocol Number:
6460

First Approval:
11/28/2011

Clinic:
MIND Clinic

Expiration Date:
10/02/2017

Contact Principal Investigator:
Matthew Milak, MD
Email: mm2354@cumc.columbia.edu
Telephone: 646-774-7526

Co-Investigator(s):
Lawrence Kegeles, MD
Dikoma Shungu, PHD
J. John Mann, MD
Michael Grunebaum, MD
M Sublette, MD, PHD
Alayar Kangarlu, PHD

Cover Sheet

Choose ONE option from the following that is applicable to your study
If you are creating a new protocol, select "I am submitting a new protocol." As 5 Year Renewals are no longer required, this option remains for historical purposes.
I am submitting an annual continuation without modifications

Division & Personnel

Division

What Division/Department does the PI belong to?
MIND
Within the division/department, what Center or group are you affiliated with, if any?
Brain Imaging

Unaffiliated Personnel

List investigators, if any, who will be participating in this protocol but are not affiliated with New York

State Psychiatric Institute or Columbia University. Provide: Full Name, Degrees and Affiliation.

Application for Continuation of Research

Status

Current Status of Study:
All research interventions were completed. Only data analysis is ongoing.

Summary of Experiences to Date

Please provide a summary of scientific progress of the study and the experience of research participants, to date. This requirement is designed to allow for the investigator and the IRB to reassess the study's risks and benefits in terms of developments in the field, changing practice patterns, and new IRB policies and procedures.
No significant observations have been made that require modifications. Data analysis is proceeding.

Funding

Have there been any changes in funding status since the prior approval?
No
Have the principal investigator and other investigators made all required disclosures of financial interest in the study sponsor/product?
Yes

Summary

Have there been any study findings, recent literature, or untoward events occuring here or at other sites in the past year which might affect the analysis of the safety, risks or benefits of study participation?
No
Have there been any serious adverse events (serious and/or unanticipated problems involving risks to subjects or others at this site which occured in the past year)?
No
Have all study staff with a significant role in the design or implementation of the human subject components of this study received required training in human research subject protections?
Yes
Is the study covered by a certificate of confidentiality?
No

Overall Progress

Approved sample size
76
Total number of participants enrolled to date
70
Number of participants who have completed the study to date
42
Have there been any significant deviations from the anticipated study recruitment, retention or completion estimates?
No
Comments / additional information

Sample Demographics

Specify population
MDD Patients
Total number of participants enrolled from this population to date
57
Specify population #2
Healthy Volunteers
Total number of participants enrolled from this population to date
13
Gender, Racial and Ethnic Breakdown
See attached PDF in the "uploads" section.

Summary of Current Year's Enrollment and Drop-out

Number of participants who signed consent in the past year
0
Did the investigator withdraw participants from the study?
No
Did participants decide to discontinue study involvement?
No

Procedures

To create the protocol summary form, first indicate if this research will include any of the following procedures

- ✓ Psychiatric Assessment
- ✓ Collection of Biological Specimens
- ✓ Medication Trial
- ✓ Use of Placebo or Sham Treatment
- ✓ MRI

- ✓ Medication-Free Period or Treatment Washout
- ✓ Administration of Substance of Abuse
- ✓ Off-label Use of Drug or Device
- ✓ Internet-based Data Collection or Transmission

Population

Indicate which of the following populations will be included in this research

- ✓ Medically and Psychiatrically Healthy Subjects
- ✓ Adults
- ✓ Adults over 50
- ✓ Inpatients

Research Support/Funding

Will an existing internal account be used to support the project?
No
Is the project externally funded or is external funding planned?
Yes
Select the number of external sources of funding that will be applicable to this study

Funding Source #1

Is the PI of the grant/contract the same as the PI of the IRB protocol?
Yes
Select one of the following
The grant/contract is currently funded
Source of Funding
Federal
Institute/Agency
NIMH
Grant Name
The Antidepressant Action of Ketamine: Brain Chemistry
Grant Number
5R01MH093637-03
Select one of the following
Single Site
Business Office
CU
Does the grant/contract involve a subcontract?
Yes
Subcontracted?
To

Name institution(s)
Research Foundation for Mental Health (RFMH)

Study Location

Indicate if the research is/will be conducted at any of the following
✓ NYSPI
This protocol describes research conducted by the PI at other facilities/locations
No

Lay Summary of Proposed Research

Lay Summary of Proposed Research
Major depressive disorder (MDD) is a common illness, affecting over 14 million American adults each year. MDD is a leading cause of disability worldwide, and is responsible for huge workplace and healthcare costs. The several week delay between onset of treatment and improvement in MDD symptoms with currently available treatments further increases the burden of the disorder. Shortening this delay is a major unmet challenge in the treatment of MDD. Studies report that a single intravenous low dose of a drug called ketamine can bring about substantial improvement in depression in hours, even in patients that have not improved with other antidepressant treatments. Certain aspects of ketamine's drug action are fairly well understood, but the question remains of how these properties relate to antidepressant effect. Our preliminary data support the rapid antidepressant benefit from ketamine. We have used a scanner to measure the effects of ketamine on two major brain chemical transmitters and found that it causes a significant increase (more than 60%) in glutamate (Glu) and gamma aminobutyric acid (GABA) levels in the front of the brain. We hypothesize that this increase in Glu and GABA levels is responsible for the antidepressant action of the medication. Knowing how ketamine works could help to develop better medications that can be used orally and used for maintenance of the improvement seen with ketamine. The objective of the proposed dose finding study is to examine the relationship between the ketamine-induced improvement of MDD and the Glu and GABA responses to ketamine and to compare the Glu and GABA responses to ketamine in MDD and healthy subjects to better understand the pathophysiology of MDD. To achieve these aims we propose a randomized, placebo-controlled, double blind study with several different doses of ketamine. We will conduct MRI scans to measure Glu and GABA before and during the ketamine treatment.

Background, Significance and Rationale

Background, Significance and Rationale

MDD is the leading cause of disability in the US for ages 15-44. The disorder affects approximately 14.8 million American adults, or about 6.7 percent of the US population age 18 and older, with estimated total annual costs of $US 43.7 billion (1990 values). The development of effective therapies for MDD is thus an ongoing priority. A related issue has been the delay of weeks in the onset of action of currently marketed

antidepressants. Intravenous administration of single sub-anesthetic doses of ketamine as a fast-acting antidepressant is reported to be effective even in previously treatment-resistant MDD.

This promising finding has been replicated and extended in a randomized, placebo-controlled trial that also indicated that the antidepressant effect endures for about a week after administration of a single dose of ketamine. It has also been demonstrated that ketamine's antidepressant mechanism of action depends on the glutamatergic AMPA receptors and the downstream induction of the mTOR pathway, which stimulates rapid protein synthesis in synapses. This mTOR-dependent dendritic and synaptic protein synthesis has been shown to be essential for learning. The mTOR signaling pathway is present, for example, in hippocampal neurons at the synaptic region, activated by glutamatergic synaptic activity, and required for LTP and LTD expression and memory consolidation. What is less clear is how ketamine, an NMDA antagonist, achieves the activation of AMPA and mTOR. These observations have formed the basis for the major objectives of this proposal: [A] We propose to investigate the mechanism by which intravenous administration of a single sub-anesthetic dose of ketamine produces rapid antidepressant effects in MDD. Glutamate (Glu) in the brain acts on N-methyl-d-aspartate/α-amino-3-hydroxy-5-methyl-4-isoxazole propionic acid and kainate (NMDA/AMPA/KA) ionotropic receptors (iGluRs) and a family of metabotropic receptors (mGluR). Our pilot in vivo human MRS data suggest a model of the antidepressant effect of ketamine. Ketamine is a noncompetitive NMDA receptor antagonist. NMDA antagonists have been shown to have acute antidepressant activity in rats. Ketamine also induces a rapid increase in brain Glu through an unknown mechanism that enhances transmission at AMPA Glu receptors and mTOR, while ketamine blocks the NMDA receptors. AMPA and mTOR activation result in increased BDNF dendritic protein synthesis, and associated downstream effects, likely accounting for the week-long efficacy of ketamine. We hypothesize that it is this shift in the ratio of NMDA to AMPA and mTOR activation by Glu that is responsible for the antidepressant effect.

To study the relationship between Glu/GABAergic response and the degree of clinical improvement at various time points after the ketamine infusion, we will employ a doubleblind, placebo-controlled study with several different doses of ketamine and placebo. This design will establish a dose-response curve between ketamine dose, clinical response, and Glu/GABA response on MRS. The use of different doses of ketamine is expected to increase the variance in the degree of clinical improvement and therefore the ability to detect a relationship to Glu/GABA responses.

Specific Aims and Hypotheses

Specific Aims and Hypotheses
Primary hypotheses.
Aim 1: We hypothesize that administration of ketamine in drug-free MDD will induce a dose-dependent reduction in the 24-item Hamilton Depression Rating Scale (HDRS-24) scores of these patients. This hypothesis will be tested within a linear model with change in HDRS score as response, dose as predictor, and other clinical and demographic covariates included as appropriate.

Aim 2: We hypothesize that administration of ketamine in drug-free MDD will induce a dose-dependent increase in glutamate levels measured with 1H MRS. This hypothesis will be tested within a linear model

with change in glutamate level as response, dose as predictor, and other clinical and demographic covariates included as appropriate.

Aim 3: We hypothesize that administration of ketamine in drug-free MDD will induce a dose-dependent increase in GABA levels measured with 1H MRS. This hypothesis will be tested within a linear model with change in GABA level as response, dose as predictor, and other clinical and demographic covariates included as appropriate.

Aim 4: We hypothesize that the magnitude of the glutamate and GABA responses to ketamine in the same MDD sample as in Aim 1 will correlate with the degree of improvement on the 24-item Hamilton Depression Rating Scale (HDRS-24) from baseline to 24 hours after ketamine infusion. This hypothesis will be tested using partial correlation analysis, i.e., determining the correlation between change in HDRS score and change in glutamate and GABA levels.

Exploratory Aims
Exploratory Aim 1: We hypothesize that the increase in the glutamate signal measured with MRS will be greater in depressed MDD patients compared with healthy non-depressed volunteers. We hypothesize a blunted GABA response in MDD. This hypothesis will be tested within separate linear model with change in (a) glutamate level and (b) GABA level as response, respectively. Predictors will include diagnosis and dose (for the MDD subjects), and other clinical and demographic covariates included as appropriate.

Description of Subject Population

Sample #1

Specify subject population
MDD Patients
Number of completers required to accomplish study aims
76
Projected number of subjects who will be enrolled to obtain required number of completers
120
Age range of subject population
18-65

Sample #2

Specify subject population
healthy volunteers
Number of completers required to accomplish study aims
10
Projected number of subjects who will be enrolled to obtain required number of completers
15
Age range of subject population

18-65

Gender, Racial and Ethnic Breakdown
No ethnic/racial/gender group is excluded. Estimate of gender and ethnic distribution of subject sample: Since subjects will be recruited in the same fashion as we have for previous studies, we expect that the sample will be similar to those who present currently to our program.

Gender: We expect more female representation as it reflects the general population where MDD is slightly more common in women.

Ethnic: Of subjects who present to our clinic, approximately 15% are African-American, 20% are members of various Latino communities and the remainder are Caucasian.
Description of subject population
Subject population is MDD Patients and Healthy volunteers ages 18-65. No ethnic/racial/gender group is excluded.

Recruitment Procedures

Describe settings where recruitment will occur
Patients and healthy volunteers will be recruited through advertisement and patients may also come from referrals from private psychiatrists and clinics. Text of advertisements and other materials to be distributed in recruitment efforts will be submitted to the IRB for approval before they are distributed.
How and by whom will subjects be approached and/or recruited?
Subjects will be recruited through advertisements and referrals from private psychiatrists.
How will the study be advertised/publicized?
Study will be advertised on websites (clinicaltrials.gov, Craigslist, our approved study website, ketaminefordepression.info) as well as through flyers. All recruitment material will be submitted and approved by the IRB before they are distributed.
Do you have ads/recruitment material requiring review at this time?
No
Does this study involve a clinical trial?
Yes
Please provide the NCT Registration Number
NCT01558063

Concurrent Research Studies

Will subjects in this study participate in or be recruited from other studies?
Yes
Describe concurrent research involvement
1. IRB #~~5880R~~ **6879R** Neuroscience Clinical Studies Initial Evaluation (PI: Maria A. Oquendo, M.D.)
2. IRB #4815 Biological and neurocognitive measures for genetic studies of psychiatric populations (PI: Maria A. Oquendo, M.D.)

3. IRB #6381R Familial Transmission of Suicidal Behavior (PI: J. John Mann, M.D.)

Inclusion/Exclusion Criteria

Name the subject group/sub sample
MDD Patients
Create or insert table to describe the inclusion criteria and methods to ascertain them
@import url(https://irb.nyspi.org/Prism/u/formulator/CuteSoft_Client/CuteEditor/Load.ashx?type=style&file=SyntaxHighlighter.css);@import url(https://fonts.googleapis.com/css?family=Source+Sans+Pro);@import url(https://fonts.googleapis.com/css?family=Lato:400,900);@import url(https://fonts.googleapis.com/css?family=Open+Sans:300,400,700&subset=latin,latin-ext);@import url(https://fonts.googleapis.com/css?family=Gloria+Hallelujah);

**Patient Inclusion**	
**Criteria**	**Method of Ascertainment**
1. Patient suffering from a major depressive episode (MDE) as part of an MDD. Patients may be psychiatric medication-free or, if on psychiatric medications, not responding adequately.	As defined by the DSM-IV by means of the SCID I; medication history and clinical examination.
2. Patient scores at least 22 on the MADRS	Montgomery-Åsberg Depression Rating Scale (MADRS)
3. Age range 18-65 years	Interview
4. Patient is off all psychotropic and other types of drugs likely to interact with glutamate for at least 14 days before starting the study. One exception is chloral hydrate or short acting benzodiazepines for distressing anxiety or insomnia (up to 72 hours prior to each MRI scan). Patients will be off neuroleptics for 1 month and off fluoxetine for 6 weeks prior to the study.	History, chart and urine drug screen
5. Subject is likely to be able to tolerate a medication washout. Only subjects who have failed their current medication regimen will be washed off medications. A failed regimen constitutes not achieving at least	Clinical interview and assessment

partial remission after an adequate dose of antidepressant medication for at least six weeks. Medication washouts will be supervised by 4-Center or 5-South for inpatients or a MIND psychiatrist for outpatients.	
6. Female subjects of child-bearing potential must be using an acceptable method of birth control throughout the study.	Clinical interview, acceptable methods include abstinence, birth control pill, male condom, IUD, depo provera, Norplant, male sterilization, diaphragm, cervical cap, female sterilization
7. Must be enrolled in IRB #4815 (PI: Oquendo)	

Create or insert table to describe the exclusion criteria and methods to ascertain them
@import url(https://irb.nyspi.org/Prism/u/formulator/CuteSoft_Client/CuteEditor/Load.ashx?type=style&file=SyntaxHighlighter.css);@import url(https://fonts.googleapis.com/css?family=Source+Sans+Pro);@import url(https://fonts.googleapis.com/css?family=Lato:400,900);@import url(https://fonts.googleapis.com/css?family=Open+Sans:300,400,700&subset=latin,latin-ext);@import url(https://fonts.googleapis.com/css?family=Gloria+Hallelujah);

**Patient Exclusion**	
**Criteria**	**Method of Ascertainment**
1. Lifetime history of schizophrenia, schizoaffective illness, Bipolar Disorder, or psychosis.	SCID; clinical history, urine drug screen
2. First-degree relative with schizophrenia, schizoaffective disorder, or bipolar disorder if the subject is less than 33 years old (mean age of onset for schizophrenia plus two standard deviations).	Clinical interview
3. Significant uncontrolled physical illness particularly if it may affect the brain or glutamatergic system including blood dyscrasias lymphomas, hypersplenism, endocrinopathies, renal failure or severe chronic obstructive lung disease, autonomic neuropathies and active malignancy.	Medical history (by a physician), physical exam, screening lab tests (chemistry panel [SMAC 20], LFTs [included in SMAC 20], CBC, HCG test for pregnancy, urinalysis, T3, T4, TSH) (no more than 59 ml will be drawn under IRB #4815)

Subjects will be excluded for baseline hypertension (BP>140/90) or significant history of cardiovascular illness.<br><br>- Absolute neutrophil count < 1500 cells/uL<br><br>- Hemoglobin < 12 g/dL for men and < 11 g/dL for women<br><br>- Platelets < 80,000 cells/uL<br><br>- LFTs >2x upper limit of normal.	
4. Significant ECG abnormalities (e.g., Ventricular tachycardia, evidence of myocardial ischemia, symptomatic bradycardia, unstable tachycardia, second degree (or greater) AV block)	ECG
5. Lacks capacity to consent	Clinical interview.
6. Patients who are actively suicidal as defined by a suicidal ideation score of 4 or 5 or suicidal behavior score > 0 on the Columbia Suicide Severity Rating Scale (C-SSRS)] at in-person screening interview will be excluded from participating as outpatients and may only participate as inpatients if the independent inpatient treatment team agrees with the plan to enroll the patient.	Clinical interview
7. ECT within the last 3 months for this episode	History, chart review (for inpatients)
8. Pregnancy or plans to conceive during the course of study participation.<br><br>Pregnancy, abortion, or lactation, lack of effective birth control during 15 days before the scans*	Urine pregnancy test at screening to rule out pregnancy or unwilling/unable to use a medically acceptable means of birth control through the course of the study. A second urine pregnancy test will be done prior to scanning on the day of the MRI.

9. Heart pacemaker, body implant or other metal (e.g., shrapnel or surgical prostheses) in body.<br><br>Metal implants or paramagnetic objects contained within the body which may present a risk to the subject or interfere with the MR scan, as determined in consultation with a neuroradiologist and according to the guidelines set forth in the following reference book commonly used by neuroradiologists: "Guide to MR procedures and metallic objects", F. G. Shellock, Lippincott Williams and Wilkins NY 2001.	Medical history (by a physician), physical exam
10. A neurological disease or prior head trauma with evidence of cognitive impairment.	Clinical interview and medical history. For subjects who endorse a history of prior head trauma, the Trail Making A and B test will be administered at the time of screening. Subjects who score ≥ 1.5 standard deviations above the mean will be excluded from study participation.
11. Patients who are responding satisfactorily to antidepressant medications because they will not be washed-out for purposes of this study; most recent antidepressant was within 6 weeks for fluoxetine and 2 weeks for all others, due to washout necessary for MRS	History
12. Claustrophobia sufficient to preclude MRI	Clinical interview, medical history (by a physician), physical exam
13. Medicinal patch**	Clinical interview, medical history (by a physician), physical exam
14. Prior ineffective trial of, or adverse effect to, ketamine	History
15. Subjects judged unlikely to be able to tolerate a psychoactive medication washout of 14 days; daily hypnotic use (occasional use of hypnotics, i.e., less than three times a week, will be allowed, except for 24 hours prior to MRS scanning);	Clinical interview
**16.** Inadequate understanding of English	Clinical interview
**17.** IV drug use or history of ketamine	Clinical interview

use as a recreational drug ≥ 2 times or an adverse reaction to ketamine; any other drug or alcohol dependence within past 6 months.	

*For females of child-bearing age: The pregnancy test is performed during the screening procedure. Since this test cannot detect the very early stage of pregnancy (10 day period between fertilization and implantation), an effective birth control method or sexual abstinence is required during the 15 days before the scan. A second pregnancy test will be done on the day of the MRI scan.

** In case the subject does have a medicinal patch, they will be asked to remove it prior to the MRI scan session.

Inclusion/Exclusion Criteria #2

Name the subject group/sub sample
Healthy Controls
Create or insert table to describe the inclusion criteria and methods to ascertain them

Control Inclusion	
**Criteria**	**Method of Ascertainment**
1. Age 18-65	Interview
2. Physically healthy	Medical history (by a physician), physical exam, screening lab tests (chemistry panel, CBC, HCG test for pregnancy, urinalysis, TSH)
3. Absence of an Axis I diagnosis (specific phobia acceptable). Absence of Borderline Personality Disorder and Antisocial Personality Disorder.	Clinical history and interview, as well as semi-structured interviews (SCID-NP and SCID-II)
4. Not on any medications known to affect glutamatergic functioning	Clinical history and urine toxicology screen.
5. Female subjects of child-bearing potential must be using an acceptable method of birth control throughout the study.	Clinical interview, acceptable methods include abstinence, birth control pill, male condom, IUD, depo provera, Norplant, male sterilization, diaphragm, cervical cap, female sterilization
6. Must be enrolled in IRB #4815 (PI: Oquendo)	

Create or insert table to describe the exclusion criteria and methods to ascertain them

Control Exclusion

Criteria	Method of Ascertainment
1. First degree relative with MDD; first degree relative with Schizophrenia, Schizoaffective Disorder, Bipolar disorder, if the subject is less than 33 years old, and therefore still at significant risk	Clinical interview
2. Significant active physical illness particularly if it may affect the brain or glutamatergic system including blood dyscrasias lymphomas, hypersplenism, endocrinopathies, renal failure or severe chronic obstructive lung disease, autonomic neuropathies and active malignancy.<br><br>Subjects will be excluded for baseline hypertension (BP>140/90) or significant history of cardiovascular illness.<br><br>- Absolute neutrophil count < 1500 cells/uL<br>- Hemoglobin < 12 g/dL for men and < 11 g/dL for women<br>- Platelets < 80,000 cells/uL<br>- LFTs >2x upper limit of normal.	Medical history (by a physician), physical exam, screening lab tests (chemistry panel [SMAC 20], LFTs [included in SMAC 20], CBC, HCG test for pregnancy, urinalysis, T3, T4, TSH) (no more than 59 ml will be drawn under IRB#4815)
3. Significant ECG abnormalities (e.g., Ventricular tachycardia, evidence of myocardial ischemia, symptomatic bradycardia, unstable tachycardia, second degree (or greater) AV block)	ECG
4. Pregnancy or plans to conceive during the course of study participation<br><br>Pregnancy, abortion, or lactation, lack of effective birth control during 15 days before the scans*	Urine pregnancy test at screening to rule out pregnancy or unwilling/unable to use a medically acceptable means of birth control through the course of the study. A second urine pregnancy test will be done prior to scanning on the day of the MRI.
5. Heart pacemaker, body implant or other metal	Medical history (by a physician), physical exam

(e.g., shrapnel or surgical prostheses) in body.<br><br>Metal implants or paramagnetic objects contained within the body which may present a risk to the subject or interfere with the MR scan, as determined in consultation with a neuroradiologist and according to the guidelines set forth in the following reference book commonly used by neuroradiologists: "Guide to MR procedures and metallic objects", F. G. Shellock, Lippincott Williams and Wilkins NY 2001.	
6. A neurological disease or prior head trauma with evidence of cognitive impairment.	Clinical interview and medical history. For subjects who endorse a history of prior head trauma, the Trail Making A and B test will be administered at the time of screening. Subjects who score ≥ 1.5 standard deviations above the mean will be excluded from study participation.
7. Claustrophobia sufficient to preclude MRI	Clinical interview, medical history (by a physician), physical exam
8. Medicinal patch**	Clinical interview, medical history (by a physician), physical exam
9. Inadequate understanding of English	Clinical interview
10. Lifetime history of substance dependence, current or past (within past 5 years) substance abuse will be excluded; IV drug use or history of ketamine use as a recreational drug ≥ 2 times or an adverse reaction to ketamine will be excluded.	Clinical interview

*For females of child-bearing age: The pregnancy test is performed during the screening procedure. Since this test cannot detect the very early stage of pregnancy (10 day period between fertilization and implantation), an effective birth control method or sexual abstinence is required during the 15 days before the scan. A second pregnancy test will be done on the day of the MRI scan.

** In case the subject does have a medicinal patch, they will be asked to remove it prior to the MRI scan session.

Waiver of Consent/Authorization

Indicate if you are requesting any of the following consent waivers
Waiver of consent for use of records that include protected health information (a HIPAA waiver of

Authorization)
No
Waiver or alteration of consent
No
Waiver of documentation of consent
No
Waiver of parental consent
No

Consent Procedures

Is eligibility screening for this study conducted under a different IRB protocol?
Yes
Indicate NYSPI IRB #
5880R, 4815
Describe Study Consent Procedures
Consent Procedure for MDD Patients

a. Subject completes a phone screen, approved under IRB #~~5880R~~ **6879R**. If the subject appears eligible based on the phone screen, they will be scheduled for an in-person screening visit under Screening Protocol (IRB #5880R) to determine whether inclusion/exclusion criteria (excluding medical screening) are met.
b. Subject is given a detailed, verbal explanation of this study (IRB #6460) and IRB #4815 and given a copy of both consent forms.
c. Subject gives written informed consent to participate in IRB #4815 and IRB #6460.
d. Subject receives medical evaluation under IRB #4815 and an ECG under IRB #6460 to rule out exclusionary medical illnesses.
e. At screening, subject will complete the MADRS and C-SSRS.

Consent Procedure for Healthy Controls

a. Subject responds to advertisement and completes a phone screen, approved under IRB #~~5880R~~ **6879R**. If the subject appears eligible based on the phone screen, they will be scheduled for an in-person screening visit under Screening Protocol (IRB #5880R) to determine whether inclusion/exclusion criteria (excluding medical screening) are met.
b. Subject is given a detailed, verbal explanation of this study (IRB #6460) and IRB #4815 and given a copy of both consent forms.
c. Subject gives written informed consent to participate in IRB #4815 and IRB #6460.
d. Subject receives medical evaluation under IRB #4815 and an ECG under IRB #6460 to rule out exclusionary medical illnesses.
e. At screening, subjects will complete the MADRS.

Indicate which of the following are employed as a part of screening or main study consent procedures

✓ Consent Form

Persons designated to discuss and document consent

Select the names of persons designated to obtain consent/assent
Grunebaum, Michael, MD
Lan, Martin, MD
Mann, J. John, MD
Milak, Matthew, MD
Miller, Jeffrey, MD
Sublette, M, MD
Type in the name(s) not found in the above list

Independent Assessment of Capacity

You have indicated that your study involves subjects who MAY LACK capacity to consent.
Does this study require an independent assessment of capacity?
No

Study Procedures

Describe the procedures required for this study

Patients:

1. Screening:

   a. We will recruit inpatients or outpatients with a major depressive episode. Patients are generally referred by their own physician or from a clinic.

   b. Subject completes a phone screen, approved under IRB **#6897R** ~~5880R~~. If the subject appears eligible based on the phone screen, they will be scheduled for an in-person screening visit under Screening Protocol (IRB **#6897R** ~~5880R~~) to determine whether inclusion/exclusion criteria (excluding medical screening) are met.

   c. Subject is given a detailed, verbal explanation of this study (IRB #6460) and IRB #4815 and given a copy of both consent forms.

   d. Subject gives written informed consent to participate in IRB #4815 and IRB #6460.

e. Subject receives medical evaluation under IRB #4815 and an ECG under IRB #6460 to rule out exclusionary medical illnesses.

f. At screening, subject will complete the MADRS and C-SSRS.

g. Subjects who are found to be eligible for study participation, but are currently taking psychiatric medication will complete a medication washout. Otherwise, patients will be monitored weekly in-person and on the telephone during the time that their MRI scan is being scheduled.

h. Weekly assessments will include Clinical Global Impressions (CGI) scale. If a patient's score on the Global Improvement section of the CGI is a six ("much worse") or seven ("very much worse") **for two consecutive CGI assessments**, the clinician ~~will~~ **may** take the patient off the protocol and initiate open clinical treatment **at the discretion of the treatment team**. Patients may independently elect to withdraw from standardized treatment at any time for any reason, independent of their CGI Global Improvement score. In addition, there is 24-hour coverage for emergencies by the Molecular Imaging and Neuropathology Division on-call physician.

2. Wash-out:

-

a. Medication washout is performed as an inpatient at NYSPI or as an outpatient by a psychiatrist in the Department of Molecular Imaging and Neuropathology. Patients will only be accepted into this study if they are either currently medication-free or they have failed an adequate trial of their current medication.

b. At a minimum, outpatients will be monitored weekly in-person and on the telephone during the washout period. Weekly assessments during washout period will include Clinical Global Impressions (CGI) scale. If a patient's score on the Global Improvement section of the CGI is a 6 ("much worse") or 7 ("very much worse") on one occasion during the washout period and the treating clinician assesses that the patient cannot safely continue research participation, the clinician will take the patient off the protocol washout and initiate open clinical treatment. If (after consultation with the research team) the clinician judges that the patient appears safe to continue research participation, the patient will continue the washout period and a repeat CGI will be done within 3-4 days. If the Improvement score remains a 6 or 7, the patient will be withdrawn from the study and continue to open treatment. Patients may

independently elect to withdraw from standardized treatment at any time for any reason, independent of their CGI Global Improvement score. In addition, there is 24-hour coverage for emergencies by the Molecular Imaging and Neuropathology Division on-call physician.

c. Patients who can only be studied safely as inpatients will be admitted to 4-Center or 5-South.

3. Treatment Plan Discussion:

a. We will dedicate a scheduled patient visit between the time of enrollment and the first ketamine/placebo infusion to formulating and discussing the details of the psychopharmacological plan post-ketamine/placebo infusion.

4. Actively Suicidal Patients:

a. Patients with a suicidal plan or intent will only be enrolled as inpatients if the independent treatment team on the inpatient research unit agrees that this is clinically reasonable. For example, if antipsychotics are indicated, the patient will not be enrolled.

b. In cases where a subject becomes too unstable during the taper or medication-free phase, as assessed by the research and/or inpatient clinical teams, to wait for the MRI session, then open clinical treatment will be initiated. If a participant is unable to do the MRI scan for any reason, then they will be dropped from the study and given open clinical treatment. Because the possible medication regimens that patients are on are myriad, it is difficult to operationalize the taper process further. For patients who require hospitalization because of destabilization or suicidal risk, admission to the NYSPI unit will be available. Hospitalized patients will be discharged from the hospital when stable as judged by the inpatient staff and the treating psychiatrist as not being in imminent risk of harm to self/other.

c. Emergency Procedures.

i. A research psychiatrist will be available by cellphone 24 hours a day, seven days a week. Patients requiring urgent admission will be brought to the CUMC Emergency Dept. by the study physician with security assistance, if

needed. Non-emergent admissions will be arranged by the treating psychiatrist, if possible to the 4-Center or 5-South. Patients who are deemed to require hospitalization, but who refuse, will receive all necessary interventions such as contacting the local crisis team, family, or Emergency Medical Services.

5. <u>MRS and MRI Scanning:</u>

-

a. Subjects will be informed that they should arrive to the clinic fasting (12 hours no food, 4 hours no liquids) prior to the ketamine infusion/MRI.

b. We will obtain MRI scans before and during a slow intravenous infusion of saline solution with ketamine hydrochloride (Abbott Laboratories, North Chicago, IL) over the course of approximately 40 minutes (see Figure 1).

c. Ketamine dosing*:*

   i. Patients will be assigned to one of five ketamine doses or placebo, as detailed in Figure 2 (p. 11).

   ii. Randomization will be performed by the study statistician, who will assign MDD participants to randomized treatment in two strata (chronic and non-chronic). Because chronicity of depression has been strongly associated with relapse after antidepressant treatment in previous studies, participants will be stratified according to chronicity. “Chronic” will be defined as current episode of major depressive disorder $\geq$ 2 years; all others will be “non-chronic.”

d. To avoid multiple sticks drawing the blood samples for ketamine, norketamine and GABA at 90 minutes and 120 minutes after the start of the ketamine infusion, a second venous line will be placed.

e. During the ketamine infusion, vital signs (blood pressure and heart rate) will be monitored as follows:

   i. -5 minutes

   ii. 0 (start of infusion)

   iii. Post start of infusion: 5, 10, 15, 20, 25, 30, 35, and 40 minutes (end of infusion)

iv. Post end of infusion:

1. Every 5 minutes for remaining duration of the scan.

2. Blood pressure will continue to be obtained until there are two measurements at least 15 minutes apart that are within 10 mmHg of the baseline diastolic blood pressure or diastolic blood pressure is at least below 85.

During the ketamine infusion and MRI scans, the vital signs are obtained through an In Vivo® machine which displays in the room with the MRI scanner and physician who supervises the patient's medical state, and also displays in the operator's room on another console (where notes can be taken). The Research Assistant who remains in the operator's room during the infusion records the blood pressure and heart rate. After the subject is transferred back to the examination room, the blood pressure and heart rate will be obtained manually by the nurse.

f. Intervention for Hypertension: If the systolic blood pressure increases to ≥ 200 or diastolic blood pressure increases to ≥115 mmHg during the ketamine infusion, the infusion will be permanently discontinued. The blood pressure will be monitored and if there is no decrease after 15 minutes, then:

i. One dose of sublingual nitroglycerine, 0.3 mg, will be administered.

ii. If there is no response within 10 minutes, clonidine 0.1 mg po will be administered every 30 minutes (total maximum dose 0.6 mg clonidine) until the desired blood pressure is reached. Desired blood pressure is defined as within normal range or 10 mmHg of baseline diastolic reading.

g. If high blood pressure is symptomatic, i.e., blurred vision, headache, chest pain, the subject will be transferred to the ER. If they do not respond to the above treatment (f) then they will be transferred to the ER.

h. Subjects will receive two MRI scans, one before and one during the administration of ketamine. MRI scans will be performed at the NYSPI MRI facility using the 3.0 Tesla GE scanner. The first measurement will consist of quantitative MRI imaging for optimal segmentation of brain tissue into gray and white matter and cerebrospinal fluid. The second measurement will be the MR spectroscopy acquisition with GABA and glutamate spectroscopic editing. Patients will receive a structural and spectroscopy MRI scan lasting approximately 90 minutes. Structural scans will include T1-weighted images.

i. Optional 2nd Ketamine Infusion for Non-Responders:

a. The ketamine response will be assessed on Days 1 and 3. Patients who maintain their non-responder status by Day 3 (defined as less than a 50% improvement in their HDRS score) following the ketamine/placebo infusion, will have the option of completing a second ketamine infusion in the Biological Studies Unit of 0.5 mg/kg, *if the ketamine dose they received during the first scan was below 0.5 mg/kg.* Non-responders who received 0.5mg/kg of ketamine during the ~~first~~ scan and who experienced no psychomimetic or cardiovascular effects requiring intervention will have the option of completing a second ketamine infusion in the Biological Studies Unit of 0.7mg/kg. However, if the patient becomes a responder at any point prior to the scheduled date of the optional 2nd scan/ketamine infusion, the 2nd ketamine infusion will be cancelled.

-

ii. Randomization and Partial Unblinding Process:

a. The study statistician performs the randomization and a physician (not involved in patient's treatment or post-ketamine ratings) will write the drug order.

b. The physician responsible for writing the drug order will also complete a form indicating the eligibility based on the randomization assignment as either 0.5 mg/kg of ketamine or less (note: the other dose will not be specified so that partial blinding is maintained.) This form will be placed in a sealed envelope to be opened only for patients who are found to be non-responders.

c. In the event that a subject remains a non-responder by Day 3, the treating physician, following the completion of the study ratings, will open the envelope to determine if the patient is eligible for the 2nd ketamine infusion of 0.5 mg/kg or 0.7mg/kg.

d. The second infusion will take place at the soonest possible date, within 1 week of the first infusion/MRI.

e. In the event that a patient elects to undergo a second ketamine infusion, the procedures will be consistent with the first infusion/MRI with the following exceptions:

i. The ketamine dose administered will be open-label 0.5 mg/kg or 0.7mg/kg.

ii. No blood samples will be drawn post-infusion. iii. No MRI scan will be conducted. The infusion will be administered under the supervision of a medical doctor and nurse in the Biological Studies Unit, following all non-MRI related safety protocols of the first infusion/scan.

6. Safety Follow-up for Ketamine Abuse/Use Follow-up:

   a. On dates equivalent to Day 1, 3 and 7, and weeks 6 and 12 post infusion, the clinician will evaluate patients using the HDRS-24, CGI, C-SSRS, BDI, and POMS and patients will complete the QIDS-SR, to assess an intent-to-treat sample.

   b. At 3 and 6 months post-ketamine, all subjects we can contact will be evaluated to determine the absence of post-exposure ketamine use/abuse.

   c. For those subjects that undergo a second or third ketamine infusion and MRI scan, they will complete a day 1, 3, and 7 follow-up for each scan, and follow-up timepoints at week 6, and months 3 and 6 will follow the final MRI scan.

   d. Outside of the mandatory follow-ups, for those who elect to take advantage of the open treatment offered, visits will occur at least every 2 weeks prior to response, and a minimum of once a month following response.

7. Withdrawal from Study:

   Subjects will be withdrawn from the study if:

   a. They request it for any reason

   b. The PI judges that it is medically unwise to continue in the study, for example if the subjects are unable to comply with the study procedures and rules.

   c. They are unable to tolerate the medication washout because of pronounced worsening of symptoms such as marked agitation, inability to drink fluids, suicidal behavior or suicidal ideation with a plan or intent.

   d. A rise in systolic blood pressure ≥ 200 mm Hg or diastolic blood pressure to ≥ 115 mm Hg during the ketamine infusion.

e. Other criteria for discontinuation will be appearance of psychosis, new suicidal ideation, mania, severe agitation, or other deterioration where the treating physician decides that a change in treatment or hospitalization is indicated.

Healthy Volunteers:

1. Screening:

a. Subject responds to advertisement and completes a phone screen, approved under IRB **#6879R** ~~#5880R~~. If the subject appears eligible based on the phone screen, they will be scheduled for an in-person screening visit under Screening Protocol (IRB **#6879R** ~~#5880R~~) to determine whether inclusion/exclusion criteria (excluding medical screening) are met.

b. Subject is given a detailed, verbal explanation of this study (IRB #6460) and IRB #4815 and given a copy of both consent forms.

c. Subject gives written informed consent to participate in IRB #4815 and IRB #6460.

d. Subject receives medical evaluation under IRB #4815 and an ECG under IRB #6460 to rule out exclusionary medical illnesses.

e. At screening, subjects will complete the MADRS.

2. MRS and MRI Scanning:

-

a. Subject will be informed that they should arrive to the clinic fasting (12 hours no food, 4 hours no liquids) prior to the ketamine infusion/MRI.

b. We will obtain MRI scans before, and during, a slow intravenous infusion of saline solution with 0.5 mg/kg of ketamine hydrochloride (Abbott Laboratories, North Chicago, IL) over the course of approximately 40 minutes (see Figure 1).

c. To avoid multiple sticks drawing the blood samples for ketamine, norketamine and GABA at 90 minutes and 120 minutes after the start of the ketamine infusion, a second venous line will be placed.

d. During the ketamine infusion, vital signs (blood pressure and heart rate) will be monitored as follows:

   i. -5 minutes

   ii. 0 (start of infusion)

   iii. Post start of infusion: 5, 10, 15, 20, 25, 30, 35, and 40 minutes (end of infusion)

   iv. Post end of infusion:

      1. Every 5 minutes for remaining duration of the scan.

      2. Blood pressure will continue to be obtained until there are two measurements at least 15 minutes apart that are within normal range or 10 mmHg of the baseline diastolic blood pressure or diastolic blood pressure is at least below 85.

During the ketamine infusion and MRI scans, the vital signs are obtained by an In Vivo® machine which displays in the room with the MRI scanner and physician who supervises the patient's medical state, and also displays in the operator's room on another console (where notes can be taken). The Research Assistant who remains in the operator's room during the infusion records the blood pressure and heart rate. After the subject is transferred back to the examination room, the blood pressure and heart rate will be obtained manually by the research assistant or nurse.

e. Intervention for Hypertension: Same as above for patients.

f. If high blood pressure is symptomatic, i.e., blurred vision, headache, chest pain, the subject will be transferred to the ER.

g. Subjects will receive two MRI scans, one before and one during the administration of ketamine. MRI scans will be performed at the NYSPI MRI facility using the 3.0 Tesla GE scanner. The first measurement will consist of quantitative, structural MRI imaging for optimal segmentation of brain tissue into gray and white matter and cerebrospinal fluid. The second measurement will be the MR spectroscopy acquisition with GABA and glutamate spectroscopic editing. Patients will receive a structural and spectroscopy MRI scan lasting approximately 90 minutes. Structural scans will include T1-weighted images.

3. Follow up: At 3 and 6 months post-ketamine, all subjects we can contact will be evaluated to determine the absence of post-exposure ketamine use/abuse.

4. Withdrawal from Study:

-

Subjects will be withdrawn from the study if:

a. They request it for any reason

b. The PI judges that it is unwise to continue in the study, for example if the subjects are unable to comply with the study procedures and rules.

c. A rise in systolic blood pressure ≥ 200 mm Hg or diastolic blood pressure to ≥ 115 mm Hg during the ketamine infusion.

d. Other criteria for discontinuation will be appearance of symptoms meeting criteria for a psychiatric disorder according to DSM-IV criteria.

You can upload charts or diagrams if any

Criteria for Early Discontinuation

Criteria for Early Discontinuation

MDD Patients

Subjects will be withdrawn from the study if:
a. They request it for any reason
b. The PI judges that it is medically unwise to continue in the study, for example if the subjects are unable to comply with the study procedures and rules.
c. They are unable to tolerate the medication washout because of pronounced worsening of symptoms such as marked agitation, inability to drink fluids, suicidal behavior or suicidal ideation with a plan or intent.

d. A rise in systolic blood pressure ≥ 200 mm Hg or diastolic blood pressure to ≥ 115 mm Hg during the ketamine infusion.
e. Other criteria for discontinuation will be appearance of psychosis, new suicidal ideation, mania, severe agitation, or other deterioration where the treating physician decides that a change in treatment or hospitalization is indicated

Healthy Controls
Subjects will be withdrawn from the study if:
a. They request it for any reason
b. The PI judges that it is unwise to continue in the study, for example if the subjects are unable to comply with the study procedures and rules.
c. A rise in systolic blood pressure ≥ 200 mm Hg or diastolic blood pressure to ≥ 115 mm Hg during the ketamine infusion.
d. Other criteria for discontinuation will be appearance of symptoms meeting criteria for a psychiatric disorder according to DSM-IV criteria.

Blood and other Biological Samples

Please create or insert a table describing the proposed collection of blood or other biological specimens
a) Protocol # 4815: Medical screening and genetics: 59 mL
b) MRI/MRS Scans: Venous line samples will be used to measure blood levels of ketamine, norketamine and plasma GABA at 90 min and 120 minutes after the start of the infusion: (2 X 16 mL for one MRI/infusion)
c) Total for MDD Responders to MRI/infusion: 91 mL
Total for Healthy Volunteers: 91 mL

Assessment Instruments

Create a table or give a brief description of the instruments that will be used for assessment

	Maximum Time (Mins)	Screening	Pre-MRI Scan	At about 80, 95, 110 Min Post Start of Infusion	~230 Min Post Start of Infusion	Follow up Days 1, 3 & 7, Weeks 6 & 12 (Month 3)[a]
**Applies to (Patients/Controls)**		**Both**	**Both**	**Both**	**Both**	**Patients**
**Hamilton Depression Rating Scale-24**	10		X			X
**Hamilton Depression Rating Scale-18**	10				X	
**The Profile of Mood States (POMS)**	1		X	X	X	X

**Beck Depression Inventory**	**10**		**X**		**X**	**X**
**Brief Psychiatric Rating Scale I**	**10**		**X**		**X**	
**MADRS**	**10**	**X**				
**C-SSRS**	**15**	**X**	**X**		**X**	**X**
**QIDS-SR**	**5**					**X**
**Clinical Global Impressions (CGI)**	**1**					**X**
**Total Maximum Time (Min)**		**25**	**46**	**1**	**46**	**42**

a. **For those subjects that undergo a second or third ketamine infusion and MRI scan, they will complete a day 1, 3, and 7 follow-up for each scan unless superseded by the following MRI scan/infusion, and follow-up timepoints at week 6 and months 3 follow the final MRI scan.**

Please attach copies, unless standard instruments are used

Off label and investigational use of drugs/devices

Choose from the following that will be applicable to your study
✓ Drug
Select the number of drugs used in this study
1

Drug #1

Name of the drug
Ketamine
Manufacturer and other information
JHP Pharmaceuticals (Ketalar)
Approval Status
No IND is required
Choose one of the following options
FDA has determined that IND is not required

Research Related Delay to Treatment

Will research procedures result in a delay to treatment?
Yes
Maximum duration of delay to any treatment
For depressed patients who are on medications that may interfere with the study when identified, other than: neuroleptics (1 month), fluoxetine (6 weeks), and serotonin-depleting drugs (3 months) (see inclusion criterion #5), the delay will not exceed 30 days (one week taper, two weeks drug-free, and one week to schedule and complete biological testing). The 30-day period takes into consideration all types of medications, tapering requirements, the drug-free period and the time required for all biological testing.

The exclusion above, of neuroleptics, fluoxetine, and serotonin-depleting drugs prior to participation will remain, but we will only include patients whose recent use of these drugs have already been terminated several weeks prior to their presentation to the investigators so that no individual should need to be maintained off medication more than 30 days before the administration of the research medication.
Maximum duration of delay to standard care or treatment of known efficacy
We expect that both the first and second ketamine infusion will be conducted within the 30-day time frame described above. We will reserve standing MRI slots to help make this possible.

The maximum delay before initiating treatment of known efficacy will be 50 days. This 40-day time period includes the maximum delay preceding the second ketamine infusion for initial non-responders (37 days), an additional 3 days after the second ketamine infusion when response will be assessed.
Treatment to be provided at the end of the study
After study completion, patients will be offered up to six months of outpatient medication treatment or up to 8 weeks of inpatient treatment or combination of inpatient and outpatient treatment in the Division of Molecular Imaging and Neuropathology clinic. Subjects who are unable to tolerate the medication washout have the option of remaining in open treatment with their assigned MIND physician for up to 6 months.

Clinical Treatment Alternatives

Clinical treatment alternatives
This is a study of intravenous ketamine in the treatment of MDE. A variety of different pharmacological (e.g. selective serotonin reuptake inhibitors, noradrenergic and dopaminergic medications, monoamine oxidase inhibitors and tricyclic antidepressants), psychotherapeutic, electroconvulsive and other therapies are available for MDE.

Risks/Discomforts/Inconveniences

Risks that could be encountered during the study period
Risks associated with participation in this study are related to 1) drug-free interval, 2) side effects of intravenous ketamine, 3) intravenous catheters, 4) blood sampling, 5) MRI scanning, 6) pregnancy, and 7) electrocardiogram.
Describe procedures for minimizing risks
5.1. Drug-free interval

We will recruit inpatients or outpatients with a major depressive episode. Patients are generally referred by their own physician or from a clinic. Medication washout is performed as an inpatient at the 4-Center or 5-South or as an outpatient by a psychiatrist in the Department of Molecular Imaging and Neuropathology. At a minimum, outpatients will be monitored weekly in person and on the telephone during the washout period. In addition, there is 24-hour coverage for emergencies by the Molecular Imaging and Neuropathology on-call physician. Only patients who currently have failed an adequate trial for their illness will be tapered off these medications. For example, patients on sub-therapeutic doses of antidepressants or in the early phase of an antidepressant trial will not be tapered, rather they will be referred back to the treating clinician with a recommendation to complete an adequate trial of medicine.

There is a risk that drug washout will result in a worsening of the patient's condition. If the responsible physician (inpatient or outpatient) determines that treatment cannot be delayed safely or the patient withdraws from the study, all necessary treatment is offered. Patients are offered up to 3mg of Ativan ≤ 2 times a week to relieve some symptoms during this phase. Ativan will be stopped for the 24 hours prior to scanning.

If the subject clinically deteriorates in the course of the drug washout to the extent that they feel they cannot tolerate the washout, or they are observed objectively to become significantly more depressed or develop suicidal ideation such that admission to hospital or medication is required clinically, we will drop them from the protocol and commence immediate appropriate treatment. In carrying out these studies over the last 10 years in depressed inpatients, many of whom were highly suicidal, no patient has completed suicide and only one patient attempted suicide during the medication washout period.

5.2. Side Effects of intravenous ketamine

Risks of ketamine injection include both medical and psychiatric or behavioral risks.

Medical risks
Administration of sub-anesthetic doses of ketamine I.V. may induce a modest rise in blood pressure. We have administered sub-anesthetic doses of ketamine I.V. (0.5mg/kg over 40 minutes) in the setting of a currently approved MRI/MRS brain imaging protocol at this institution (IRB #5786, PI: J. Mann). The resulting effects on vital signs for the eleven patients scanned under protocol #5786 are presented as a function of time in Table 1 below for the duration of the ketamine injection. These modest increases all peaked and largely resolved by 75 minutes, with vitals returning to near baseline.

Specific measures and precautions
Any medical risks from increased blood pressure will be minimized through the careful screening of potential subjects. Subjects will be excluded for baseline hypertension or any history of cardiovascular illness. In addition, an ACLS certified physician, Dr. Mate Milak, Dr. Michael Grunebaum **or** Dr. Lawrence Kegeles ~~or Dr. Carolyn Rodriquez,~~ will be present during the procedure. Nausea and vomiting will be treated supportively and, if severe, with anti-emetic agents; if necessary, administration of ketamine will be discontinued. Subject will be informed that they should be fasting (12 hours no food, 4 hours no liquids) prior to the ketamine infusion/MRI. For these reasons, the medical risks involved in participation in this study will be minimized.

Table 1. Effects of ketamine on systolic and diastolic blood pressure in a group of patients (n=11). The dose of ketamine was 0.5 mg/kg given over 40 minutes.

Time (min)	Systolic Blood Pressure during Ketamine (mm Hg)	Diastolic Blood Pressure during Ketamine (mm Hg)	Pulse during Ketamine (min^{-1})
**0**	111	70	69
**5**	113	72	63
**10**	116	73	69

**15**	116	73	72
**20**	121	75	68
**25**	121	78	73
**30**	120	77	72
**35**	124	78	73
**40**	122	78	79
**45**	122	78	74
**50**	116	75	73
**55**	121	76	74
**60**	123	76	75
**65**	124	79	75
**70**	121	81	76
**75**	114	75	76

Psychiatric or behavioral risks
Ketamine is an FDA-approved dissociative anesthetic. Ketamine exposure at the sub-anesthetic dose to be used in this study can be associated with a moderate dissociative state, which is well tolerated in the majority of cases and spontaneously reversible.1 There is extensive clinical experience with ketamine used at anesthetic doses and no long-term detrimental effects of ketamine exposure have been reported. It is possible that ketamine administration will increase the risk of psychosis, even in normal subjects. Ketamine is a street drug of abuse, sometimes called 'special k.' As such, it poses the risk that exposure during this study may predispose subjects to subsequent abuse of this drug. For this reason, a careful history of any past or present problems with substance abuse will be obtained on screening and any such positive history will constitute an exclusion criterion. Moreover, we will follow patients while they are on open treatment and review any evidence of abuse that may appear after the ketamine infusion.

Specific measures and precautions
The experiment will be carried out in the presence of at least one psychiatrist. Severe agitation or hyperarousal will be treated with intravenous benzodiazepine (lorazepam) or neuroleptics, as indicated. The risks of exposing subjects to a drug of abuse potential will be minimized by explaining this risk to prospective subjects, and by excluding from the study any subjects with documented or suspected prior substance or alcohol dependence history. Any subjects requiring admission due to any possible adverse outcomes related to the administration of ketamine will be cleared by a physician for discharge from either 4-Center or 5-South the morning after ketamine administration.

5.3. Intravenous catheters

There is a small risk of infection and bleeding associated with intravenous catheters, which are prevented by proper techniques. Placement of IVs will be by a physician or nurse.

5.4. Blood sampling

Adverse effects of blood sampling will be minimized by exclusion of subjects with low hemoglobin levels during screening (Hemoglobin < 12 g/dl in men and < 11 g/dL in women).

5.5. Discomfort during scanning

It may be uncomfortable to lie motionless in the scanner (MRI) and it may cause some subjects to feel anxious. Our staff will be available to provide support, reduce anxiety, optimize the comfort of the subject and remove the subject from the machine if requested.

5.6. MRI scanning

While there have been no reports of any long-term ill effects caused by magnets of the same or even higher strength, the long-term effects of being placed in a magnet of this strength (3 Tesla) are unknown. The MRI scanner uses a large magnet to take pictures of the brain and is not associated with any known medical risks, except for persons who have a heart pacemaker, or have metal in their body (e.g. shrapnel or surgical prostheses) which may be affected by the magnet. Patients will be asked to notify us if this is the case. There is also the risk of burns from medicinal patches during the MRI; therefore, subjects will be asked to remove any patches prior to the scanning session. Also, although there are no known risks associated with pregnancy, we will not scan anyone who is pregnant. Therefore, for women of childbearing years, pregnancy testing will be conducted the day of the MRI. Some people have reported sensations during the MRI scan such as “tingling” or “twitching” (or, very rarely a painful sensation), which are caused by changes in the magnetic field that can stimulate nerves in your body. If the subject experiences sensations and feels uncomfortable, the MR technologist will stop the scan immediately. Occasionally, some people experience nervousness or claustrophobic feelings due to the scanner’s small space. Despite these sensations, in our experience, no one has had sensations from the scanning that did not stop as soon as the scanning stopped. The MRI scan is not painful, but having to lie still in the enclosed space of the scanning table is uncomfortable for some people.

5.7. Pregnancy

Women of child-bearing age will be required to have a urine pregnancy test administered at the time of initial screening as well as within 24 hours prior to the MRI scan. Subjects will not be charged for the pregnancy test. They are excluded partly because of unknown risk to the fetus and because lying in the scanner may be difficult and there may be biological effects on the brain of fetus.

5.7. Electrocardiogram

An electrocardiogram has no serious risks. On rare occasions a rash may develop where the electrodes are places which usually resolves without treatment.

6. Describe benefits to subjects, if any.

Subjects will receive a complete medical, neurological and psychiatric evaluation, results of which will be communicated to them. Subjects are not expected to benefit directly from participation in this study; however, the results of the MRI will be shared with the subject and/or a physician of their choice. Because this MRI scan is being performed for research purposes only it may not show problems that would normally

be found in a typical clinical, diagnostic MRI scan ordered by a doctor for a specific medical problem.

7. Confidentiality: Describe means by which privacy will be protected and confidentiality of data maintained. Include procedures for the storage and protection of electronic data. NOTE: If a Certificate of Confidentiality will be obtained for this study, please indicate.

Once a subject enrolls in the project they are given a code number, which is used for all subsequent data and/or lab forms. The code list and subject names as well as all data are kept in locked files with access limited to those directly responsible for maintenance of these files by the research team or institutional personnel as part of a routine audit.

The MRI report will be maintained as part of the clinical database at the New York State Psychiatric Institute along with the subject's name and will be accessible to clinicians. For patients (only), their psychiatric diagnosis will not be part of the report. All subjects will receive feedback about their scans. All results will be shared with research subjects in a manner that is consistent with the acuity and certainty of the finding, and will be communicated by an appropriately qualified member of the research team in the form of a letter approved by the IRB and will be contacted by telephone if there is evidence of an abnormality. Results will be shared with the subject or a physician designated by the subject.

Methods to Protect Confidentiality

Describe methods to protect confidentiality
Once a subject enrolls in the project they are given a code number, which is used for all subsequent data and/or lab forms. The code list and subject names as well as all data are kept in locked files with access limited to those directly responsible for maintenance of these files by the research team or institutional personnel as part of a routine audit.

The MRI report will be maintained as part of the clinical database at the New York State Psychiatric Institute along with the subject's name and will be accessible to clinicians. For patients (only), their psychiatric diagnosis will not be part of the report. All subjects will receive feedback about their scans. All results will be shared with research subjects in a manner that is consistent with the acuity and certainty of the finding, and will be communicated by an appropriately qualified member of the research team in the form of a letter approved by the IRB and will be contacted by telephone if there is evidence of an abnormality. Results will be shared with the subject or a physician designated by the subject.
Will the study be conducted under a certificate of confidentiality?
No

Direct Benefits to Subjects

Direct Benefits to Subjects
Subjects will receive a complete medical, neurological and psychiatric evaluation, results of which will be communicated to them. Subjects are not expected to benefit directly from participation in this study; however, the results of the MRI will be shared with the subject and/or a physician of their choice. Because

this MRI scan is being performed for research purposes only it may not show problems that would normally be found in a typical clinical, diagnostic MRI scan ordered by a doctor for a specific medical problem.

Compensation and/or Reimbursement

Will compensation or reimbursement for expenses be offered to subjects?
Yes
Please describe and indicate total amount and schedule of payment(s).
Include justification for compensation amounts and indicate if there are bonus payments.
Subjects will receive $150.00 for completion of the MRI/MRS scans and the ketamine infusion, $50 for the baseline evaluation, and $50 for the post-treatment assessments, for a total of $250. For non-responders that elect to undergo a second infusion, there will be not be any compensation for the second infusion.

References

References

1. **Krystal JH, Karper LP, Seibyl JP, et al. Subanesthetic effects of the noncompetitive NMDA antagonist, ketamine, in humans. Psychotomimetic, perceptual, cognitive, and neuroendocrine responses. *Arch Gen Psychiatry.* Mar 1994;51(3):199-214.**

2. **Dakwar E, et al. The effects of subanesthetic ketamine infusions on motivation to quit and cue-induced craving in cocaine-dependent research volunteers. *Biol Psychiatry.* Jul 2014; 76(1):40-46.**

Uploads

Upload the entire grant application(s)
Upload copy(ies) of unbolded Consent Form(s)
Upload copy(ies) of bolded Consent Form(s)
Upload copy(ies) of the HIPAA form
6460_HIPAA_9_23_15.pdf
Upload any additional documents that may be related to this study
6460_ACAR_2015_recruitment_demo_final Sheet1.pdf

Patients

Ethnic Category	Sex/Gender		
	**Females**	**Males**	**Total**
Hispanic or Latino	4	3	7
Not Hispanic or Latino	29	21	50
Unknown (individuals not reporting ethnicity)	0	0	0
**Ethnic Category: Total of All Subjects***	33	24	57
**Racial Categories**			
American Indian/Alaska Native	0	0	0
Asian	2	4	6
Native Hawaiian or Other Pacific Islander	0	0	0
Black or African American	1	6	7
White	27	11	38
More Than One Race	3	1	4
Unknown or Not Reported	0	2	2
**Racial Categories: Total of All Subjects***	33	24	57

Controls

Ethnic Category	Sex/Gender		
	**Females**	**Males**	**Total**
Hispanic or Latino	1	0	1
Not Hispanic or Latino	6	6	12
Unknown (individuals not reporting ethnicity)	0	0	0
**Ethnic Category: Total of All Subjects***	6	6	12
**Racial Categories**			
American Indian/Alaska Native	0	0	0
Asian	0	0	0
Native Hawaiian or Other Pacific Islander	0	0	0
Black or African American	2	3	5
White	2	4	6
More Than One Race	2	0	2
Unknown or Not Reported	0	0	0
**Racial Categories: Total of All Subjects***	6	7	13

New York State Psychiatric Institute (NYSPI)
Authorization to Use or Disclose Health Information during a Research Study

Protocol Number: 6460 **Principal Investigator:** Mate I. Milak, M.D.

Name of Study: The Antidepressant Action of Ketamine: Brain Chemistry

Before researchers can use or share any identifiable health information ("Health Information") about you as part of the above study (the "Research"), the New York State Psychiatric Institute (NYSPI) is required to obtain your authorization. You agree to allow the following individuals and entities to use and disclose Health Information about you as described below:

- New York State Psychiatric Institute (NYSPI), your doctors and other health care providers, if any, and
- The Principal Investigator and his/her staff (together "Researchers"). Researchers may include staff of NYSPI, the New York State Office of Mental Health (OMH), Research Foundation for Mental Hygiene, Inc. (RFMH), and Columbia University (CU), provided such staff is a part of the study, and
- Providers of services for the Research at CU, NYSPI and/or RFMH, such as MRI or PET, or Central Reference Laboratories (NKI), if indicated in the consent form**.**

1. **The Health Information that may be used and/or disclosed for this Research includes:**

☑ All information collected during the Research as told to you in the Informed Consent Form.
☑ Health Information in your clinical research record which includes the results of physical exams, medical and psychiatric history, laboratory or diagnostic tests, or Health Information relating to a particular condition that is related to the Research.
☐ Additional information may include:

2. **The Health Information listed above may be disclosed to:**

☑ Researchers and their staff at the following organizations involved with this Research:
Cornell Medical College, Citigroup Biomedical Imaging Center

☐ The Sponsor of the Research,

and its agents and contractors (together, "Sponsor"); and
☑ Representatives of regulatory and government agencies, institutional review boards, representatives of the Researchers and their institutions to the level needed to carry out their responsibilities related to the conduct of the research.
☐ Private laboratories and other persons and organizations that analyze your health information in connection with this study

☐ Other (family members or significant others, study buddies, outside agencies etc.) Specify:

3. By giving permission to release your Health Information as described above, you understand that your Health Information may be disclosed to individuals or entities which are not required to comply with the federal and state privacy laws which govern the use and disclosure of personal Health Information by NYSPI. This means that once your Health

Information has been disclosed to a third party which does not have to follow these laws (e.g., a drug company or the Sponsor of the Research), it may no longer be protected under the HIPAA or NYS Mental Hygiene Law requirements but is subject to the terms of the consent form and may be subject to other state or federal privacy laws or regulations.

4. **Please note that:**
   - You do not have to sign this Authorization form, but if you do not, you may not be able to participate in the study or receive study related care. You may change your mind at any time and for any reason. If you do so, you may no longer be allowed to participate in the study. If you withdraw this Authorization the research staff and the Sponsor, if this is sponsored research, may still use or disclose Health Information containing identifying information they already have collected about you as needed to maintain the reliability of the research. Any request to withdraw this Authorization must be made in writing to (enter name and contact information below):

     Mate I. Milak, M.D.
     New York State Psychiatric Institute
     1051 Riverside Drive, Box 42
     New York, NY 10032

   - While the Research is going on, you may not be allowed to review the Health Information in your clinical research record that has been created or collected by NYSPI. When this research has been completed you may be allowed to see this information. If it is needed for your care, your Health Information will be given to you or your Doctor.

5. **This Authorization does not have an end date.**

6. **You will be given a copy of this form after you have signed it.**

I agree to the use and disclosure of Health Information about me as described above:

____ ______________________

Signature of Participant/ Legal Representative Date

____

Printed Name of Participant

____

Relationship of Legal Representative to Participant (if applicable)

We also ask you or your legal representative to initial the statements below:

☐ **I have received a copy of the NYSPI/OMH Notice of Privacy Practices.**
