# Supplementary material for: Assessment of Relationship of Ketamine Dose With Magnetic Resonance Spectroscopy of Glx and GABA Responses in Adults With Major Depression: A Randomized Clinical Trial
Source: JAMA Netw Open. 2020 Aug 12;3(8):e2013211. doi: 10.1001/jamanetworkopen.2020.13211 (PMC7424409; doi:10.1001/jamanetworkopen.2020.13211)
Supplement: Supplement 2. — eFigure 1. Proton Magnetic Resonance Spectroscopy (1H-MRS) Example Voxel Placement and J-Edited Spectra eFigure 2. Plasma Concentration of Ketamine eFigure 3. Change in Glx and GABA Levels Following Initiation of Ketamine Infusion, Corrected for Internal Water and Expressed as a Percentage of Preketamine Glx eTable. Serious Adverse Events Occurring During Study Participation [file jamanetwopen-3-e2013211-s002.pdf]

Supplementary Online Content

Milak MS, Rashid R, Dong Z, et al. Assessment of relationship of ketamine dose with magnetic resonance spectroscopy of Glx and GABA responses in adults with major depression: a randomized clinical trial. *JAMA Netw Open*. 2020;3(8):e2013211. doi:10.1001/jamanetworkopen.2020.13211

eFigure 1. Proton Magnetic Resonance Spectroscopy (1H-MRS) Example Voxel Placement and J-Edited Spectra
eFigure 2. Plasma Concentration of Ketamine
eFigure 3. Change in Glx and GABA Levels Following Initiation of Ketamine Infusion, Corrected for Internal Water and Expressed as a Percentage of Preketamine Glx
eTable. Serious Adverse Events Occurring During Study Participation

This supplementary material has been provided by the authors to give readers additional information about their work.

© 2020 Milak MS et al. *JAMA Network Open.*

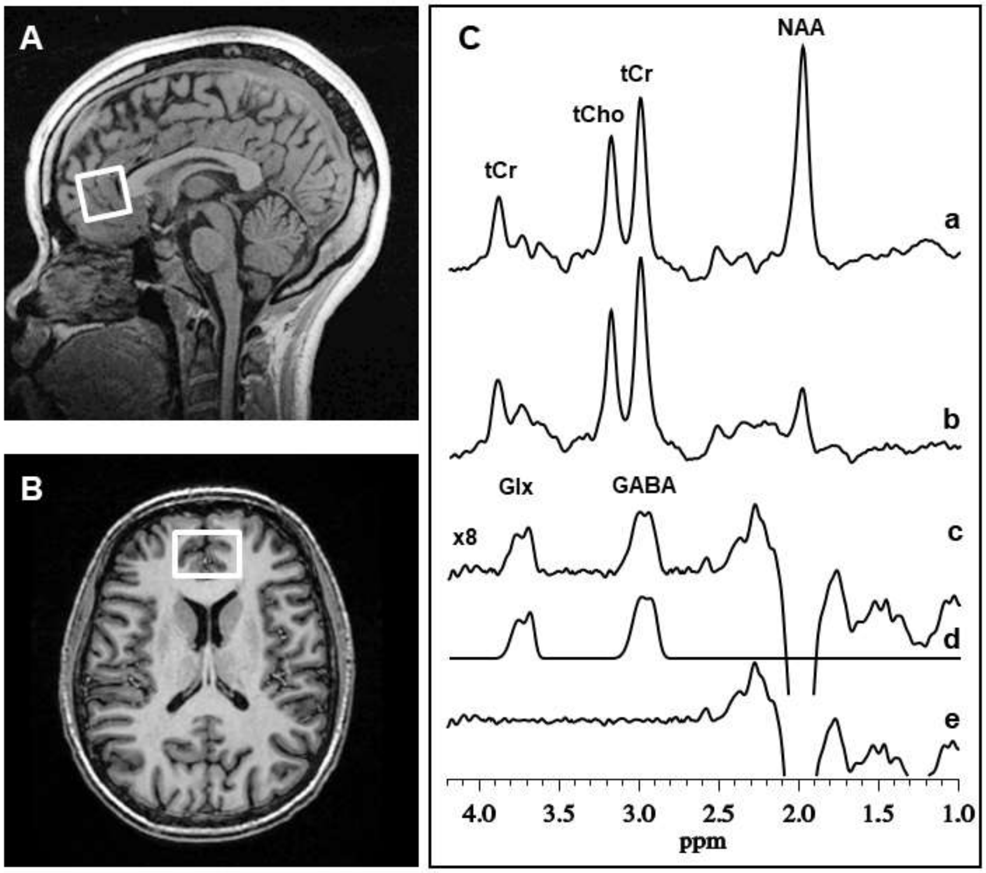

eFigure 1. Proton Magnetic Resonance Spectroscopy (^{1}H MRS) Example Voxel Placement and J-Edited Spectra

(A) Sagittal and (B) axial localizer images showing the size and location of the medial prefrontal cortex (mPFC) voxel of interest. (C) Demonstration of in vivo human GABA and Glx detection by ^{1}H MRS in the voxel of interest: (a and b), single-voxel subspectra acquired in about 13 min with the editing pulse on and off and 256 (512 total) interleaved averages; spectrum (c), difference between spectra (a and b) showing the edited brain GABA and Glx resonances; spectrum (d), model-fitting of the experimental spectrum (c) to obtain the GABA and Glx peak areas; spectrum (e), residual of the difference between spectra (c and d). GABA, γ-aminobutyric acid; Glx, glutamate+glutamine; NAA, N-acetyl-aspartate; tCho, total choline; tCr, total creatine.

© 2020 Milak MS et al. *JAMA Network Open.*

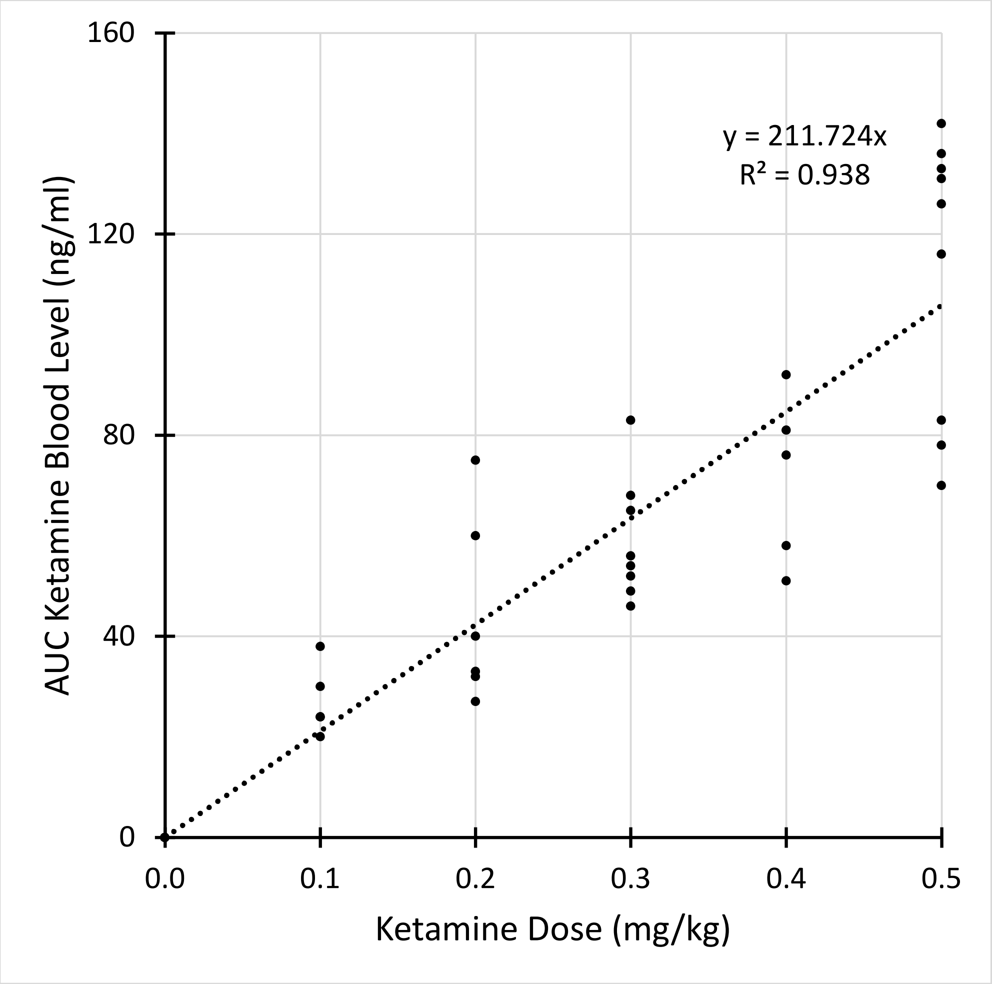

eFigure 2. Plasma Concentration of Ketamine

Measured as area under the curve (AUC) between 90 and 120 minutes following initiation of ketamine infusion, correlation with ketamine dose. Each dot represents a single subject.

© 2020 Milak MS et al. *JAMA Network Open.*

(**A**)

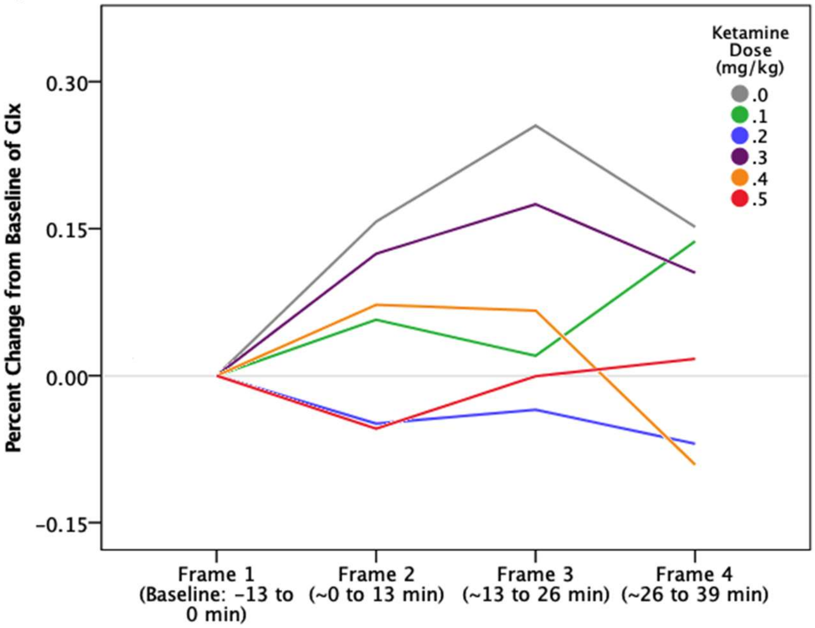

(**B**)

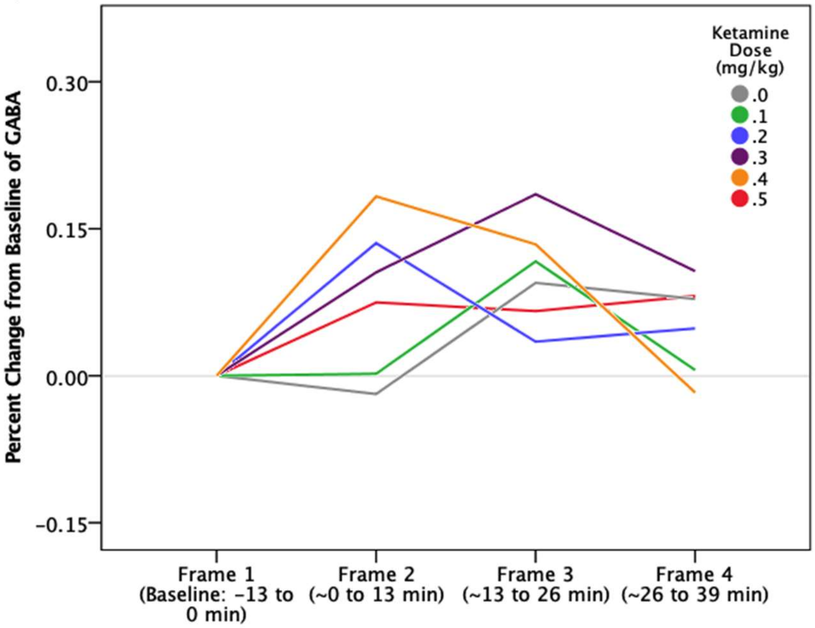

eFigure 3. Change in Glx (A) and GABA (B) Levels Following Initiation of Ketamine Infusion, Corrected for Internal Water and Expressed as a Percentage of Preketamine Glx

© 2020 Milak MS et al. *JAMA Network Open.*

eTable. Serious Adverse Events Occurring During Study Participation

Adverse events were checked by an active but general inquiry during study participation and outpatient follow-up. Side effects and adverse events were assessed immediately post-infusion and again at 24 hours post-infusion. Many patients were in our clinic for 6 months of free medication treatment after the study was complete and general examination revealed further medical issues, that were considered to be unrelated to the intervention. One subject took an overdose of their previous medication before the ketamine infusion, and that was clearly not a consequence of the infusion. Of note, aside from the psychotomimetic effects which are analyzed in detail, other significant side effects including vomiting, rise in blood pressure requiring intervention, etc., did not occur in this study.

**Serious adverse event (SAE)**	**Time since ketamine infusion**
Death by suicide.	~5 months
Active suicidal ideation.	~6 weeks
Antidepressant misuse: bupropion overdose.	Pre-ketamine infusion
Unrelated medical illness: Leg injury due to fainting spell.	~3 months

© 2020 Milak MS et al. *JAMA Network Open.*
