# Supplementary material for: Assessment of Relationship of Ketamine Dose With Magnetic Resonance Spectroscopy of Glx and GABA Responses in Adults With Major Depression: A Randomized Clinical Trial
Source: JAMA Netw Open. 2020 Aug 12;3(8):e2013211. doi: 10.1001/jamanetworkopen.2020.13211 (PMC7424409; doi:10.1001/jamanetworkopen.2020.13211)
Supplement: Supplement 3. — Data Sharing Statement [file jamanetwopen-3-e2013211-s003.pdf]

Data Sharing Statement

Milak. Assessment of Relationship of Ketamine Dose With Magnetic Resonance Spectroscopy of Glx and GABA Responses in Adults With Major Depression. *JAMA Netw Open*. Published August 12, 2020. 10.1001/jamanetworkopen.2020.13211

Data
Data available: Yes
Data types: Deidentified participant data
How to access data: Requests for data should be sent to jjm@columbia.edu
When available: beginning date: 02-01-2021

Supporting Documents
Document types: None

Additional Information
Who can access the data: See above
Types of analyses: For statistical analysis for relevant papers.
Mechanisms of data availability: After approval of a proposal, or with a signed data access agreement
